# Identification of the global regulatory roles of RraA via the integrative transcriptome and proteome in *Vibrio alginolyticus*

**DOI:** 10.1128/msphere.00020-24

**Published:** 2024-06-27

**Authors:** Huizhen Chen, Qian Gao, Bing Liu, Ying Zhang, Jianxiang Fang, Songbiao Wang, Youqi Chen, Chang Chen

**Affiliations:** 1 South China Sea Institute of Oceanology, CAS Key Laboratory of Tropical Marine Bio-Resources and Ecology (LMB), South China Sea Institute of Oceanology, Chinese Academy of Sciences, Guangzhou, China; 2 College of Earth and Planetary Sciences, University of Chinese Academy of Sciences, Beijing, China; 3 College of Marine Sciences, South China Agricultural University, Guangzhou, China; 4 Guangzhou College of Technology and Business, Guangzhou, China; 5 Xisha Marine Environmental National Observation and Research Station, Sansha, China; NC State University, Raleigh, North Carolina, USA

**Keywords:** *Vibrio alginolyticus*, RraA, polymyxin B, biofilm formation, transcriptome and proteome, metabolism

## Abstract

Bacterial ribonuclease E (RNase E) is vital for posttranscriptional regulation by degrading and processing RNA. The RraA protein inhibits RNase E activity through protein-protein interactions, exerting a global regulatory effect on gene expression. However, the specific role of RraA remains unclear. In this study, we investigated *rraA* expression in *Vibrio alginolyticus* ZJ-T and identified three promoters responsible for its expression, resulting in transcripts with varying 5′-UTR lengths. During the stationary phase, *rraA* was significantly posttranscriptionally inhibited. Deletion of *rraA* had no impact on bacterial growth in rich medium Luria-Bertani broth with salt (LBS) but resulted in decreased biofilm formation and increased resistance to polymyxin B. Transcriptome analysis revealed 350 differentially expressed genes (DEGs) between the wild type and the *rraA* mutant, while proteome analysis identified 267 differentially expressed proteins (DEPs). Integrative analysis identified 55 genes common to both DEGs and DEPs, suggesting that RraA primarily affects gene expression at the posttranscriptional level. KEGG (Kyoto Encyclopedia of Genes and Genomes) analysis demonstrated that RraA facilitates the conversion of fatty acids, propionic acid, and branched-chain amino acids to acetyl-CoA while enhancing amino acid and peptide uptake. Notably, RraA positively regulates the expression of virulence-associated genes, including those involved in biofilm formation and the type VI secretion system. This study expands the understanding of the regulatory network of RraA through transcriptome analysis, emphasizing the importance of proteomic analysis in investigating posttranscriptional regulation.

**IMPORTANCE:**

RraA is an inhibitor protein of ribonuclease E that interacts with and suppresses its endonucleolytic activity, thereby playing a widespread regulatory role in the degradation and maturation of diverse mRNAs and noncoding small RNAs. However, the physiological functions and associated regulon of RraA in *Vibrio alginolyticus* have not been fully elucidated. Here, we report that RraA impacts virulence-associated physiological processes, namely, antibiotic resistance and biofilm formation, in *V. alginolyticus*. By conducting an integrative analysis of both the transcriptome and proteome, we revealed the involvement of RraA in carbon metabolism, amino acid catabolism, and transport, as well as in the type VI secretion system. Collectively, these findings elucidate the regulatory influence of RraA on multiple pathways associated with metabolism and pathogenesis in *V. alginolyticus*.

## INTRODUCTION

RNA stability plays a very important role in the posttranscriptional modulation of gene expression and is affected by many pathways in bacteria, including those involving endoribonucleases, exoribonucleases, and RNA pyrophosphate hydrolases (RppH values). Ribonuclease E (RNase E) is thought to be a major player in mRNA decay and processing, in addition to being involved in the maturation of rRNA and tRNA (1). The cellular levels and activity of RNase E in bacteria are tightly controlled (2). Ribonuclease activity regulator A (RraA), a conserved 17.4 kDa protein, is a protein inhibitor that interacts with RNase E and inhibits its cleavage (3).

The interaction between RraA and RNase E leads to an overall change in RNA abundance as well as functionally altered gene expression. Gene chip analysis has shown that the mutation of RraA results in dramatic changes in the global abundance of mRNA in *Escherichia coli*, affecting more than 15% of cellular transcripts (4). The overexpression of RraA in *E. coli* increases the relative abundance of 2,000 mRNAs, which are involved primarily in envelope biosynthesis and aerobic metabolism (3). RraA can also affect multiple components of RNA degradation vesicles in a dynamic, energy-dependent manner (5).

Numerous orthologs of *E. coli* RraA, including VvRraA1 and VvRraA2 in *Vibrio vulnificus*, RraA in *Pseudomonas aeruginosa*, and ScRraA1 and ScRraA2 in *Streptomyces coelicolor*, have been found (6–12). In *S. coelicolor*, compared with the wild-type strain, the *rraAS1* gene deletion results in a greater growth rate and increased production of actinorhodin and undecylprodigiosin (11). In *V. vulnificus*, VvRraA1 modulated the pathogenicity of *V. vulnificus* in mice (12). Transcriptomic analysis indicated that VvRraA1 modulates the abundance of a large number of mRNA transcripts (12). Taken together, these findings suggest that RraA is a global regulator that controls a wide range of cellular and physiological processes in a species-specific manner. However, the ortholog of RraA in *V. alginolyticus*, a widespread pathogen of marine fish and humans, has not yet been investigated.

Here, we characterized the ortholog of RraA in *V. alginolyticus*, constructed a deletion strain, a complementary strain, and an overexpression strain of *rraA*, and subsequently evaluated their growth ability, motility, antibiotic resistance, and biofilm formation ability. In addition, the RraA regulon of *V. alginolyticus* during the early exponential growth phase in an LBS medium was investigated using integrated high-throughput RNA-seq and proteomics analysis.

## MATERIALS AND METHODS

### Bacterial strains, plasmids, and growth conditions

The bacterial strains and plasmids used in this study are listed in Table 1. *V. alginolyticus* was routinely cultured in Luria–Bertani (LB) medium supplemented with 2.5% NaCl at 30°C. *E. coli* strains were cultured in LB media supplemented with appropriate antibiotics at 37°C. Thiosulfate citrate bile salts sucrose (TCBS) medium (HuanKai, Guangzhou, China) containing 5 µg/mL chloramphenicol (Cm) and 0.2% D-glucose was used for selecting transconjugants. To identify transgenic bacteria that underwent plasmid excision and allelic exchange, the *ccdB* gene was induced using TCBS medium supplemented with 0.2% arabinose plus 5 µg/mL Cm or not, after which bacteria that had lost the inserted plasmid were selected. When necessary, the *E. coli* media were supplemented with antibiotics at the following final concentrations: ampicillin, 100 µg/mL; Cm, 20 µg/mL; and, if needed, diaminopyrimidine at a final concentration of 0.3 mM in the growth media.

**TABLE 1:** Strains and plasmids used in this study

Strain or plasmid	Relevant characteristics	Source
*Vibrio alginolyticus* strains		
ZJ-T	Ap^r^, translucent/smooth variant of wild strain ZJ51; isolated from diseased *Epinephelus coioides* off the South China coast	(13)
ZJ-T-Δ*rraA*	Ap^r^; ZJ-T carrying a deletion of *rraA*	This study
ZJ-T-Δ*rraA+*	Cm^r^; ZJ-T-Δ*rraA* carrying a *rraA* complementation plasmid pMMB207-*rraA*	This study
ZJ-T/over *rraA*-pSCT32	Cm^r^; ZJ-T carrying a RraA expression plasmid over *rraA*-pSCT32	This study
ZJ-T/pSCT32-gfp-*rraA*-TL	Cm^r^; ZJ-T carrying a *rraA* translational fusion plasmid pSCT32-gfp-*rraA*-TL	This study
*E. coli* strain		
GEB883	WT; *E. coli K12 ΔdapA::ermpir* RP4-2 Δ*recA gyrA462*, *zei*298::Tn10; donor strain for conjugation	(14)
Plasmids		
pSW7848	Cm^r^; suicide vector with an R6K origin, requiring the Pir protein for its replication, and the *ccdB* toxin gene	(15)
pSW7848-Δ*rraA*	Cm^r^; pSW848 containing the flanking region fragments in the mutant allele of *rraA*	This study
pMMB207	Cm^r^; RSF1010 derivative, *IncQ lacI*^q^ P*tac oriT*	(16)
pSCT32	Cm^r^; expression plasmid with a pBR322 and a f1 origin at the same time and a *trc* promoter	(17)
pMMB207-*rraA*	Cm^r^; pMMB207 containing the wild-type allele of *rraA*	This study
pSCT32-*rraA*	Cm^r^; pSCT32 containing the wild-type allele of *rraA*	This study
pSCT32-gfp	Cm^r^; pSCT32 containing promoterless reporter gene *gfp* encoding a green fluorescent protein	This study
pSCT32-gfp-*rraA*-TL	Cm^r^; *rraA* (including all three promoters and start codon, from −493 to +3 nt) are translationally fused to pSCT32-gfp	This study

### Sequence analysis

The species used in this analysis and their accession IDs are as follows: EcRraA (AAB01208.1AAB01208.1 *E. coli* strain_K12), StRraA (WFG57654.1 *Salmonella enterica* subsp. *enterica* serovar Typhimurium), VvRraA 1(ACK76427.1 *V. vulnificus MO6-24/O*), VvRraA 2(ACK76428.1 *V. vulnificus MO6-24/O*), VaRraA (WP_005397954.1 *Vibrio alginolyticus*), VcRraA (1VI4_A Chain A *Vibrio cholerae*)PaRraA (QPV51173.1 *P. aeruginosa*), MtRraA (ANZ84631.1 *Mycobacterium tuberculosis*), TtRraA (WVY30247.1 *Thermus thermophilus*), ScRraA1 (WMT37493.1 *S. coelicolor*), and ScRraA2 (5 × 15_A Chain A *S. coelicolor*). Sequence analysis was performed by constructing a phylogenetic tree based on amino acid differences using the maximum likelihood method with 300 bootstrap replicates. This analysis was carried out using MEGA X software (downloaded from http://www.megasoftware.net/, accessed on 6 June 2022). The Percent Identity Matrix among different strains, created by Clustal2.1, can be found at the following website: https://www.ebi.ac.uk/jdispatcher/msa/mafft.

### Construction of the mutant, complementary, and overexpression strains

The mutant strains were constructed according to Grimm et al. (2003) (18). Briefly, the upstream and downstream regions of *rraA* were obtained through PCR using the primers RraA-UP-F/R and RraA-DOWN-F/R (Table S2). Simultaneously, the vector fragment pSW7848 was amplified through PCR employing the primers pSW7848-F/R (Table S2). The recombinant suicide plasmid pSW7848-Δ*rraA* was obtained through isothermal assembly and subsequently transformed into GEB883 cells (Table 1). The complementary strain was constructed with a pMMB207-based plasmid by insertion of the *rraA* open reading frame (ORF) under the P_tac_ promoter. The *rraA* overexpression strain was constructed with the high-copy-number plasmid pSCT32, which harbors the *rraA* gene under the P_trc_ promoter. The details of transformation, recombination, and selection of target strains were performed as previously described by Liu et al. (19, 20).

### Whole-transcriptome sequencing by RNA-seq

Three single colonies of each strain were cultured overnight. The cells were then diluted 1,000-fold in LBS medium and grown to the early logarithmic phase (OD_600nm_ approximately ≈ 0.6). Subsequently, the cells were collected from 100 mL of culture. RNA was extracted using the high-purity TransZol Up Plus RNA Kit (TransGen Biotech, Beijing, China) following the extraction protocol. The purified RNA was subsequently sent to Genedenovo Biotech (Guangzhou, China) for the construction of single-end RNA-Seq libraries, which were subsequently sequenced using the Illumina NovaSeq 6000 platform with paired-end 150-base reads. The detailed data processing was carried out as previously described by Liu et al. (19).

The differentially expressed genes (DEGs) were identified based on a fold change ≥2 and a false discovery rate-adjusted *P* value (*q* value) <0.05. The DEGs were then subjected to enrichment analysis of GO (Gene Ontology) annotations and KEGG pathways using a threshold of *q* values < 0.05.

### 5'-RACE System for rapid amplification of cDNA ends

RNA extraction of wild-type ZJ-T was performed as previously described in the section “Whole-transcriptome sequencing by RNA-seq.” The primers used are listed in Table S2 and were designed with the NCBI Primer Design Tool (https://www.ncbi.nlm.nih.gov/tools/primer-blast/) (Table S2). An Evo M-MLV reverse transcription kit (Accurate Biotechnology Co., Ltd., Hunan, China) was used for cDNA synthesis, and a 5′-RACE kit (Sangon Biotech, Shanghai, China) was used for 5′-UTR amplification, PCR product purification, and tailing.

### Quantitative reverse transcription PCR analysis

RNA extraction and quantitative real-time fluorescent quantitative PCR (qRT-PCR) were performed as previously described (19). The ΔΔCt method was used to calculate relative expression levels, with *recA* serving as the reference gene (21). The experiment was repeated at least twice. The primers used for qRT-PCR are listed in Table S2, and statistical significance was assessed using Student’s *t* test (ns *P* > 0.05, *P < 0.05, and **P < 0.01).

### Growth measurement

Growth measurements in rich medium LBS from ZJ-T, ZJ-T-Δ*rraA*, ZJ-T-Δ*rraA*+, and ZJ-T/over *rraA*-pSCT32 were carried out as previously described by Liu et al. (19, 20). The experiments were repeated three times.

### Motility assay

Overnight cell cultures of ZJ-T, ZJ-T-Δ*rraA*, ZJ-T-Δ*rraA*+, and ZJ-T/over *rraA*-pSCT32 were adjusted to OD_600nm_ = 1.0 in LBS medium. Five microlitres of each culture were spotted on 0.3% agar plates and 1.5% agar plates. The plates were inverted in a 30°C incubator for 24 hours. The diameter was subsequently measured to evaluate the motility of the strains. Statistical significance was assessed using one-way analysis of variance (ANOVA) with the least significant difference (LSD) method (*P < 0.05 and **P < 0.01).

### Antibiotic sensitivity test

The antibiotic sensitivity test was carried out by using an antimicrobial drug sensitization tablet kit (Hangzhou Microbial Reagent Co., Ltd., Hangzhou, China). Briefly, single clones of *V. alginolyticus* ZJ-T, ZJ-T-Δ*rraA*, ZJ-T-Δ*rraA*+, and ZJ-T/over *rraA*-pSCT32 were inoculated into LBS liquid media and incubated overnight at 30°C with shaking at 200 rpm. Then, 200 µL of the overnight bacterial solution was mixed with 10 mL of fresh LBS liquid medium, poured onto LBS plates, and dried for 5 minutes. Antibiotic paper sheets containing different concentrations of antibiotics were added to the plates using sterile forceps, and the plates were incubated in an incubator at 30°C for 24 hours. The diameters of the inhibition zones were subsequently measured and recorded. The experiments were repeated at least three times.

### Biofilm formation assay

The overnight culture of the strains (ZJ-T, ZJ-T-Δ*rraA*, ZJ-T-Δ*rraA*+, and ZJ-T/over *rraA*-pSCT32) was adjusted to an OD_600nm_ = 1.0. Fifty microlitres of the diluted cultures were inoculated into 5 mL of LBS liquid medium and incubated in glass tubes at 30°C. The cultures were removed, and the tubes were collected 3 hours, 12 hours, 24 hours, and 48 hours postinoculation. The tubes were washed three times with distilled water, after which the matrix attached to the glass tubes was stained with 200 µL of 2% crystal violet stain in ethanol for 5 minutes. The excess stain was gently rinsed away with water, and 200 µL of pure ethanol was added to dissolve the remaining crystal violet. The solutions were pipetted into a 96-well plate, and a spectrophotometer was used to measure the absorbance at 550 nm.

### Translational fusion and analysis

To identify the promoter of *rraA*, a translational fusion based on the green fluorescent protein (GFP) reporter was constructed according to previous methods (19). In brief, fragments of different lengths from the upstream region of the *rraA* start codon, which includes the longest length and the start codon (from −493 to +3), were cloned and inserted ahead of the ORF of *gfp* in the pSCT32 vector (Table S2). The resulting plasmid was subsequently transformed into *E. coli* GEB883 receptor cells, which were confirmed via PCR analysis and sequencing.

Western blotting was used to quantify the target protein fusions. For the western blotting procedure, samples were collected at OD_600nm_ readings of 0.5, 3.0, and 5.0, all of which were cultured in LBS containing 5 µg/mL Cm. Cells from three independent biological replicates were collected, centrifuged, and resuspended in 100 µL of 2 × SDS loading buffer per OD_600nm_ unit (Sangon Biotech, Shanghai, China). Subsequently, the samples were incubated at 100°C for 10 minutes. SDS-PAGE, western blotting, and visualization were performed according to previous methods (19). The data presented represent the most representative results from three technical replicates.

## RESULTS

### Phylogenetic analysis and characterization of the *rraA* transcript of *V. alginolyticus*

**Fig 1:**
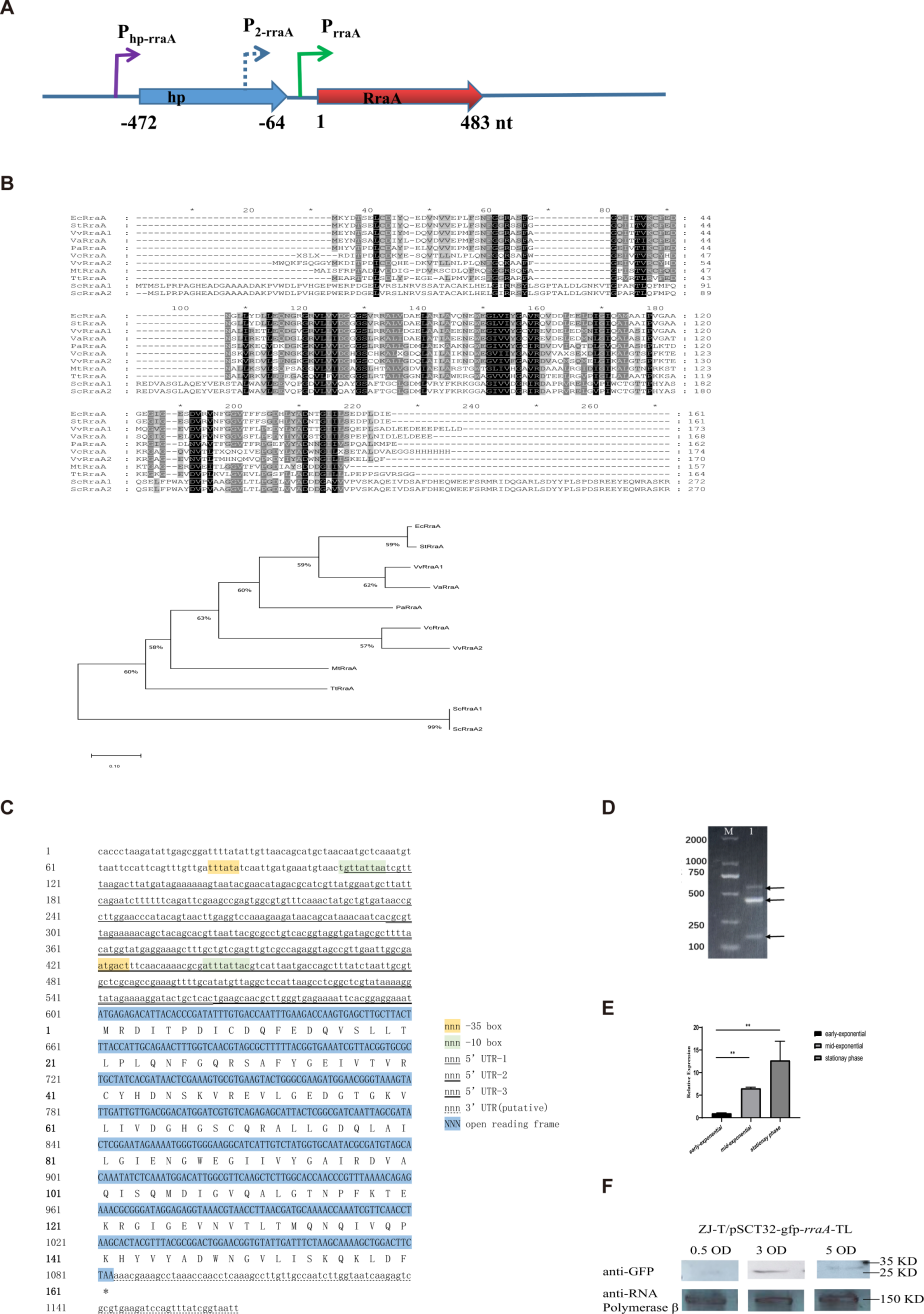
Bioinformatic analysis of the *rraA* sequences of *V. alginolyticus* and their expression in the LBS. (**A**) Schematic diagram of the *rraA* coding position. The arrows indicate the transcriptional and coding directions. (**B**) Alignment of amino acid sequences of *V. alginolyticus* RraA and its orthologs in different bacteria. (**C**) Analysis of the sequence structure and promoter of *rraA*. (**D**) 5′-RACE experiments of the *rraA* gene. (**E**) Relative expression of *rraA* at different stages (Student’s *t* test, *P* values: *, <0.05; **, <0.01). The levels of *rraA* were normalized to the expression of the housekeeping gene *recA*. Error bars represent SDs. (**F**) Western blot of the *rraA* translational fusion at three growth stages at OD_600nm_ values of 0.5, 3.0, and 5.0. Anti-RNA polymerase β as an internal reference.

The *rraA* gene is located on chromosome 1 of *V. alginolyticus* ZJ-T. A hypothetical protein (HP), separated by a 64 bp intergenic region, is located upstream of *rraA* (Fig. 1A). However, in *E. coli*, *rraA* is located downstream of the *menA* gene, which encodes a 1,4-dihydroxy-2-naphthoic acid octaprenyltransferase that catalyzes the prenylation of menaquinone (4, 22). The amino acid sequences of RraA were analyzed using the multiple sequence comparison tool MUSCLE (http://www.ebi.ac.uk/Tools/msa/muscle/), which generated a similarity matrix (percent identity matrix). The similarity between VaRraA of *V. alginolyticus* and VvRraA2 of *V. vulnificus* reached a maximum of 86.90%. Moreover, the similarities between VvRraA2 of *V. vulnificus* and VcRraA of *V. cholerae* were found to be 41.885% and 39.52%, respectively (Fig. 1B; Fig. S1). Some of the conserved polar amino acids, considered interaction sites with RNase E, may be located on the surface of the molecule. Moreover, the two cysteines, Cys9 and Cys41, are identical in orthologs of all bacteria and are considered key amino acids for the formation of RraA homohexamers (23).

The transcription of *rraA* was reported to originate from its promoter, and P*_rraA_* is located in the intergenic region in *E. coli* (4). Nevertheless, transcriptome analysis in this study revealed that *rraA* has a 493 nt-long 5′-UTR (untranslated region) that contains part of the upstream *hp* coding region, indicating that two or more promoters may be involved in its transcription in *V. alginolyticus* ZJ-T (Fig. 1C). Furthermore, 5′-RACE analysis revealed at least three transcripts with start points corresponding to −493 nt, −305 nt, and −38 nt (the A of the start codon is +1; Fig. 1D), consistent with the results of BPROM prediction. The −38 nt sequence may correspond to the conserved promoter of P*_rraA_* in *E. coli*.

A continuous increase in the *rraA* transcript abundance was observed with cell growth, as determined by quantitative RT-PCR. When the cell culture entered the stationary phase, there was a 12-fold increase compared to that in the early log phase. This observation is consistent with previous findings in *E. coli* (4) (Fig. 1E). However, there was a discrepancy between the mRNA level of *rraA* and its corresponding protein at different growth stages. While the RraA protein level gradually increased from the early to late log phase, it subsequently decreased during the early stationary phase (Fig. 1F). This finding suggested that the expression of *rraA* in *V. alginolyticus* may be significantly regulated posttranscriptionally, possibly at the translational efficiency level, during the stationary phase.

### Positive regulatory effect of RraA on polymyxin B resistance and biofilm formation in *V. alginolyticus* ZJ-T

**Fig 2:**
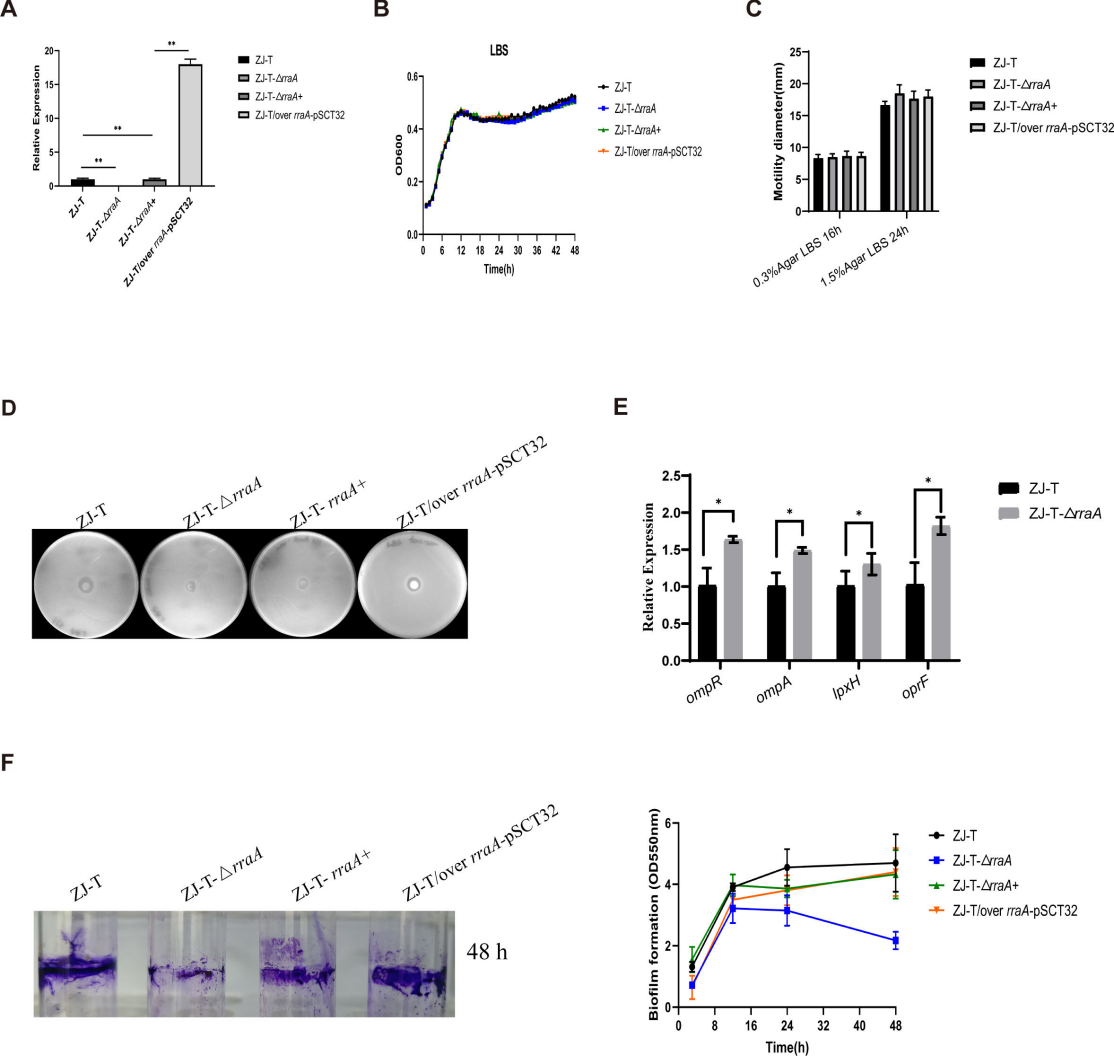
The physiological role of *rraA* in *V. alginolyticus*. (**A**) Relative expression of *rraA* in the wild-type, *rraA* mutant, complementary, and overexpression strains. (**B**) Growth curves of the wild-type, *rraA* mutant, complementary, and overexpression strains grown in LB +2.5% NaCl-rich medium. (**C**) The swimming and swarming abilities of ZJ-T, ZJ-T-Δ*rraA*, ZJ-T-Δ*rraA*+, and ZJ-T/over *rraA*-pSCT32 on 0.3% and 1.5% LBS agar plates. (**D**) Antibiotic resistance to polymyxin B in wild-type ZJ-T, ZJ-T-Δ*rraA*, ZJ-T-Δ*rraA*+, and ZJ-T/over *rraA*-pSCT32. (**E**) Relative mRNA expression levels of the outer membrane proteins, lipid A biosynthesis genes, and two-component regulator genes. The levels of genes were normalized to the internal control *recA* level. Error bars indicate SD (Student’s *t* test, *P*-value: ns, >0.05 and **, <0.01). (**F**) Biofilm synthesis of *V. alginolyticus* wild-type ZJ-T, the *rraA* mutant, the complemented strain, and the overexpression strain at different time points.

To investigate the role of *rraA* in *V. alginolyticus* ZJ-T, we constructed an in-frame deletion of *rraA* in ZJ-T, named ZJ-T-Δ*rraA*, and its complementary strain ZJ-T-Δ*rraA*+, which carried the low-copy-number plasmid pMMB207 harboring the entire coding region of *rraA* transcribed from the P*tet* promoter. Additionally, we constructed an overexpression strain, ZJ-T/over *rraA*-pSCT32, which carried the high-copy-number plasmid pSCT32 with the same construction as above. qRT-PCR revealed that, compared with that in the WT, the expression of *rraA* in the complemented strain was completely rescued, while the overexpression strain exhibited an increase in *rraA* expression of approximately 17–18-fold (Fig. 2A).

It has been reported that the overexpression of RraAV1 results in a moderately slower growth rate than that of the WT in *V. vulnificus* (7, 24). However, in the present study, changes in the *rraA* transcript abundance did not significantly affect the growth of LBS in rich media (Fig. 2B). Furthermore, no variations were observed in either swimming or swarming motility among the strains (Fig. 2C), which contrasts with the decreases in swimming and swarming motility observed in the RraAV1 mutant strain of *V. vulnificus* (12, 24).

Thirty antibiotics were used to test the antibiotic resistance of the cells. As shown in Table S1, the alteration of *rraA* resulted in no change in resistance to any of the tested antibiotics except polymyxin B (Fig. 2D). Interestingly, both the deletion and overexpression of *rraA* increased the resistance of the cells to polymyxin B (Fig. 2D). In order to further explore the susceptibility of *V. alginolyticus* to polymyxin B in the *rraA*-free background, we have studied expression levels of genes related to outer membrane proteins, lipid A biosynthesis genes, and two-component regulator genes (Fig. 2E). In strain ZJ-T-Δ*rraA,* the expression levels of *ompR*, *ompA*, *lpxH*, and *oprF* were all increased (Fig. 2E).

To evaluate the possible effect of *rraA* on biofilm formation, we quantified the biofilm formation of the four strains at different time points during growth. As shown in Fig. 2F, the biofilm formation ability of ZJ-T increased during the log phase and remained stable at its peak during the stationary phase. In comparison, the attached biomass of ZJ-T-Δ*rraA* continued to increase but was significantly less than that of ZJ-T during the log phase. Moreover, the concentration decreases as the cells enter the stationary phase. At 48 h postinoculation, biofilm formation in ZJ-T-Δ*rraA* decreased by approximately 50% (Fig. 2F). Moreover, the profiles of the complemented and overexpression strains were similar to those of the wild type. These results suggest that RraA positively regulates biofilm formation in *V. alginolyticus*.

### RraA regulon revealed by integrative transcriptome and proteome analysis

To investigate the regulatory role of RraA, we conducted high-throughput RNA-seq and DIA label-free proteomic analyses of ZJ-T and ZJ-T-Δ*rraA* cells harvested at the early log phase (OD_600 nm_=0.5).

**Fig 3:**
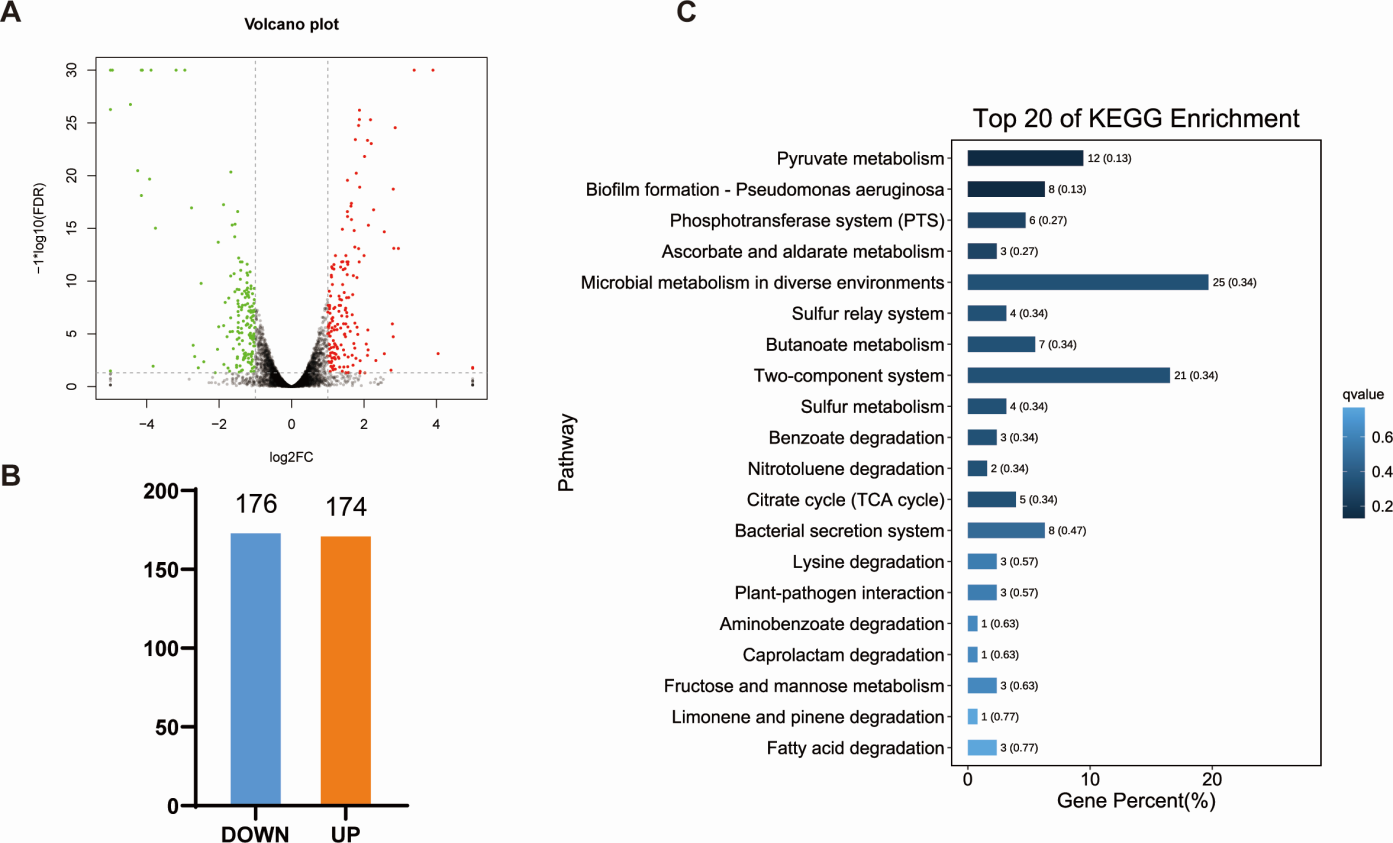
Overview of the RNA transcriptomic profiles of the wild-type and *rraA* mutant strains. (**A**) Volcano plot of differentially expressed genes (DEGs) in the transcriptome [FDR < 0.05, | log2(fold change) | ≥ 1]. Upregulated expression is displayed in red, downregulated expression is displayed in green (judged by FDR < 0.05 and differential expression greater than twofold), and black points show no differences. (**B**) Statistical column chart of DEGs. WT: wild-type strain *V. alginolyticus* ZJ-T; RraA: *rraA* mutant strain ZJ-T-Δ*rraA*. (**C**) Histogram of the enrichment of the top 20 KEGG pathways identified via transcriptomics after *rraA* inactivation.

**Fig 4:**
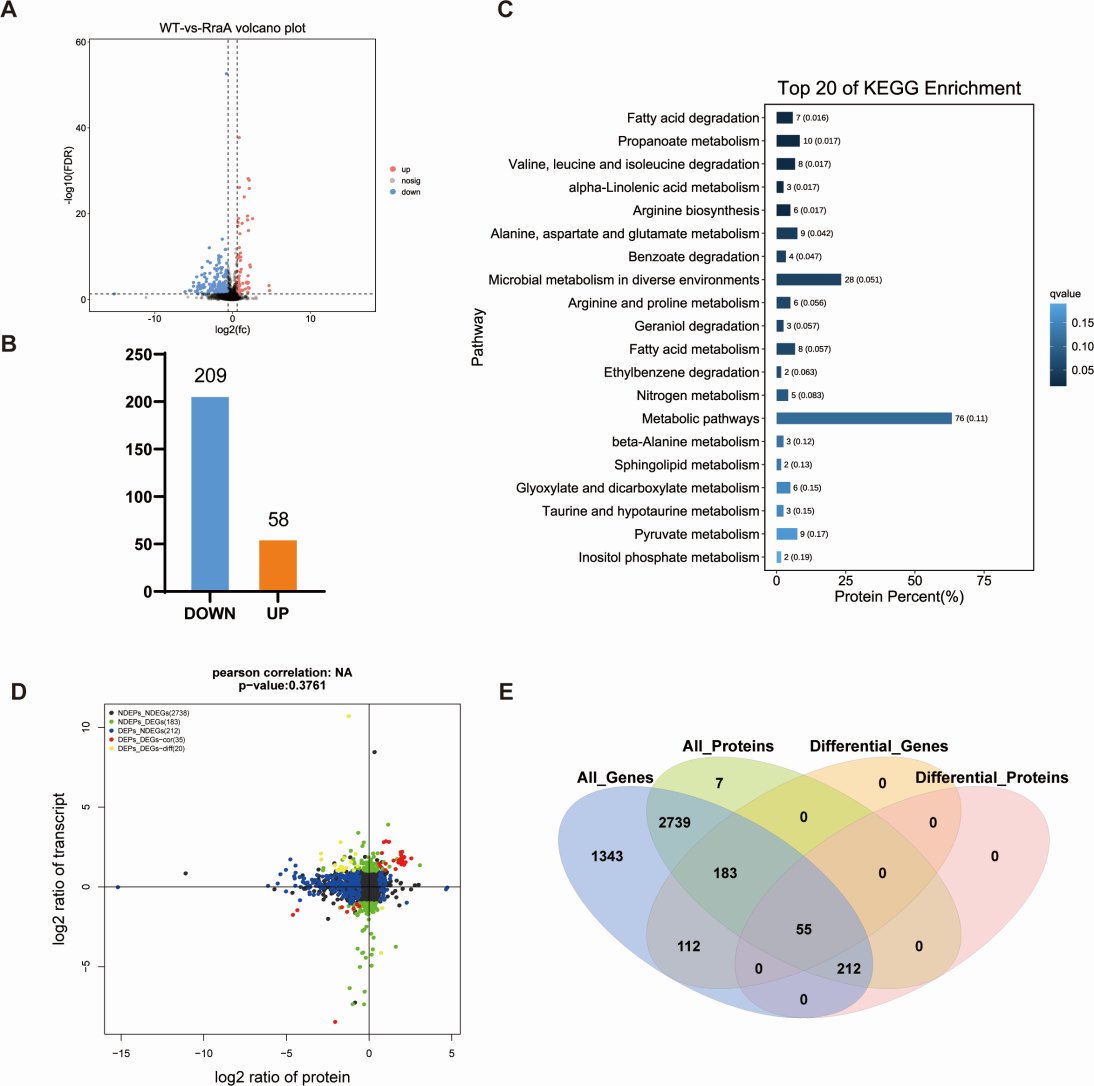
Overview of the proteomic profiles of the wild-type and *rraA* mutant strains. (**A**) Volcano plot of DEGs identified via proteomics (FDR < 0.05, |fold change| ≥ 1.5). The red dots represent upregulated differences; the blue dots represent downregulated differences; and the black dots represent no differences. (**B**) Statistical column chart of the DEPs. WT: wild-type strain *V. alginolyticus* ZJ-T; RraA: *rraA* mutant strain ZJ-T-Δ*rraA*. (**C**) Histogram of the enrichment of the top 20 KEGG pathways identified by proteomics when *rraA* was knocked out. (**D**) Venn diagram of DEGs and DEPs between the wild-type and *rraA* mutant strains. (**E**) Nine-quadrant plots of the transcriptome and proteome of the wild-type ZJ-T strain and the *rraA* mutant strain. The black dots indicate non-DEPs and genes, the red dots indicate consistent or opposite trends in gene and protein changes, the green dots indicate DEGs but non-DEPs, and the blue dots indicate non-DEGs but DEPs (if differential ploidy is reached and the p-value is not reached, the dots are shown in gray).

**Fig 5:**
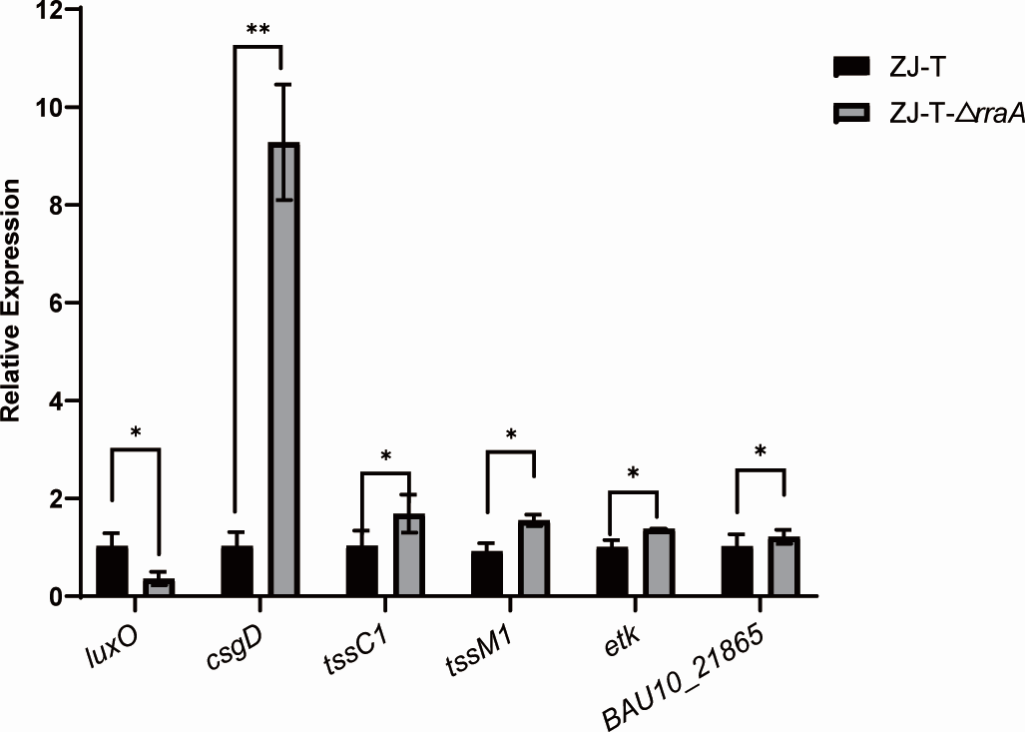
Relative expression of genes checked by qRT-PCR. The levels of genes were normalized to the internal control *recA* level. Error bars indicate SD (Student’s *t* test, *P*-value: ns, >0.05 and **, <0.01).

By comparing the ZJ-T group with the ZJ-T-Δ*rraA* group, we identified 350 DEGs in ZJ-T-Δ*rraA*, including 174 upregulated genes and 176 downregulated genes (Fig. 3A and B), with criteria set at FDR < 0.05 and |log_2_FC| > 1. All DEGs are listed in Table S3. Quantitative proteomic analysis revealed 267 differentially expressed proteins (DEPs), comprising 58 upregulated and 209 downregulated proteins, in ZJ-T-Δ*rraA* (FDR < 0.05 Fig. 4A and B). These genes and proteins were further subjected to KEGG pathway enrichment analysis (Fig. 3C and 4C ). All DEPs are listed in Table S4. To validate the transcriptome analysis, we selected five genes, *luxO* (BAU10_06930), *csgD* (BAU10_22065), *etk* (BAU10_21860), *tssC1*(BAU10_20240), and *tssM1* (BAU1020265) and BAU10_21865, to measure their transcript abundances using qRT-PCR. As shown in Fig. 5, the expression profiles are generally in agreement with the -omics data.

The combined transcriptomic and proteomic analysis revealed a total of 55 genes that were concurrently present among both the DEGs and DEPs (Fig. 4E). Additionally, 212 genes were identified exclusively as DEPs and nondifferentially expressed genes (NDEGs), while 183 genes were unique to nondifferentially expressed proteins (NDEPs) and DEGs. Furthermore, a substantial number of 2,739 genes were observed solely in the categories of NDEGs and NDEPs. Interestingly, 466 genes exhibited increased protein levels, and 192 genes exhibited decreased protein levels. The mutant had no variation in transcript abundance, suggesting that more than half of the differentially expressed genes are primarily regulated at the translational level (Fig. 4D).

### Positive regulation of fatty acid catabolism

**Fig 6:**
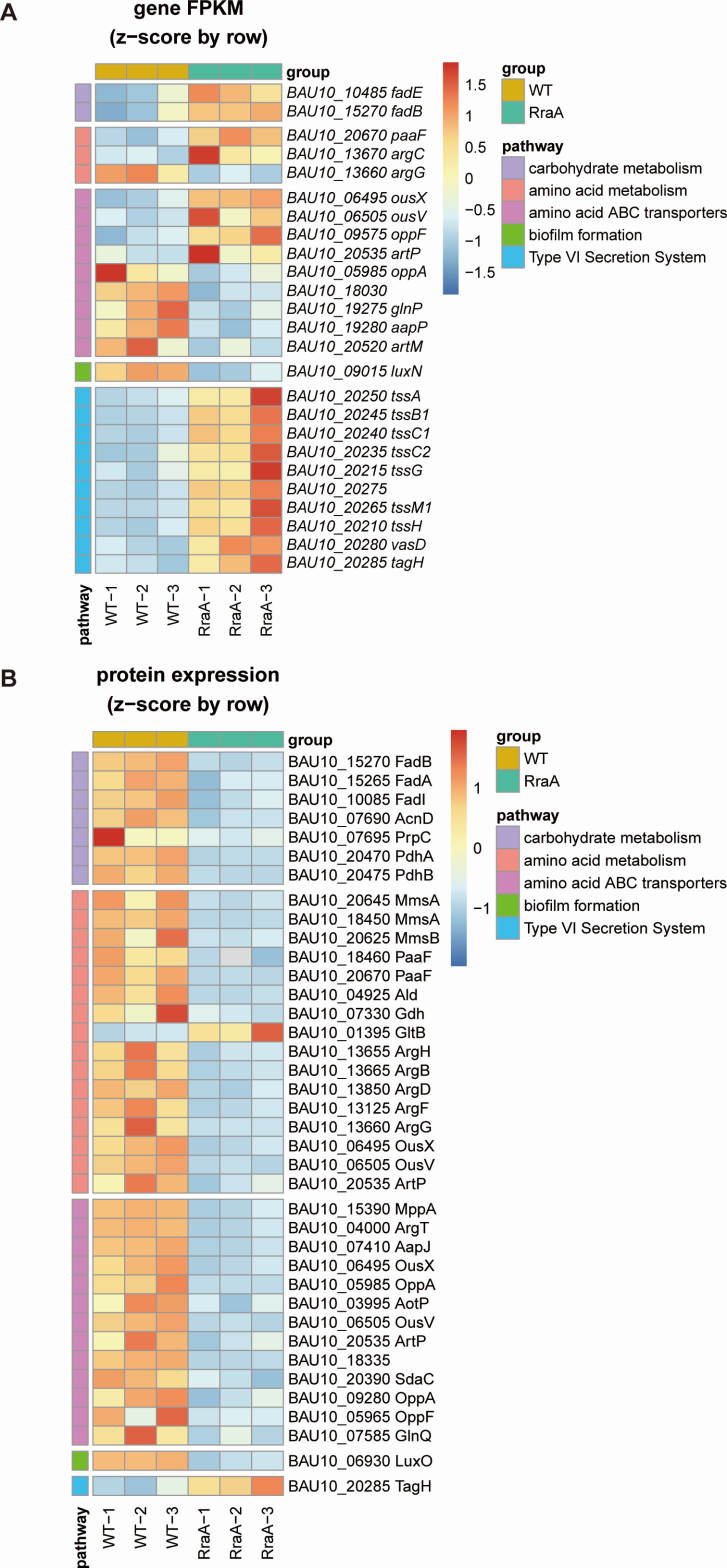
Heatmaps of DEGs and DEPs in the wild-type and *rraA* mutant strains. The pathways shown included carbohydrate metabolism, amino acid metabolism, ABC transporters, biofilm formation, and the type VI secretion system. Hierarchical clustering of samples and the relationships between genes and proteins were analyzed based on gene expression, measured as fragments per kilobase of exon model per million mapped fragments (FPKM), and protein expression data. Each column represents a sample, and each row represents a gene. The expression of genes in different samples is indicated by different colors. A redder color indicates higher expression, and a bluer color indicates lower expression.

The genes identified as DEGs or DEPs were enriched in various metabolic pathways (Fig. 3C and 4C). Notably, carbohydrate pathways such as those related to the degradation of fatty acids and propionic acid were significantly affected. The degradation of fatty acids is mediated by β-oxidation, which involves enzymes such as FadI (acetyl-CoA C-acyltransferase), FadD (ATP-dependent acyl-CoA synthase), FadE (acyl-CoA dehydrogenase), FadB (3-hydroxyacyl-CoA dehydrogenase), and FadA (3-ketoacyl-CoA thiolase) in bacteria (25–28). β-Oxidation can yield acetyl-CoA or both acetyl-CoA and propionyl-CoA, depending on the carbon number (even or odd) of the fatty acid chain. In this study, the protein abundances of FadE (BAU10_10485), FadB (BAU10_15270), FadA (BAU10_15265), and FadI (BAU10_10085) decreased by 1.67–3.17-fold at the protein level, although the abundance of fadE decreased in the mutant (Fig. 6A and B), suggesting that RraA positively regulates fatty acid degradation in *V. alginolyticus*.

Propionyl-CoA is generated through the methylcitrate cycle, which is the primary pathway for propionyl-CoA utilization in bacteria (25). In *Vibrio* species, this sequence of reactions is catalyzed by four core enzymes: methylcitrate synthase (PrpC), methylcitrate dehydratase (PrpD), aconitase (AcnD), and 2-methylaconitate cis-trans isomerase (PrpF). These enzymes are presumably encoded by the operon prpDB-acnD-prpFE, and they convert propionyl-CoA to pyruvate in a 1:1 molar ratio. Here, the levels of the proteins AcnD (BAU10_07690) and PrpC (BAU10_07695) were reduced by 2.53- and 6.01-fold, respectively, in the mutant (Table S4), indicating that impairment of RraA slows the methylcitrate cycle pathway, leading to a decrease in the pyruvate pool within the cell.

Notably, two subunits of pyruvate dehydrogenase, which is responsible for catalyzing the conversion of pyruvate to acetyl-CoA and is encoded by PdhAB (BAU10_20470 and BAU10_20475), were also significantly reduced by more than 12.17- and 22-fold, respectively, in the mutant (Table S4).

### Positive role in amino acid transport and metabolism

Branched-chain amino acids (BCAAs) are initially converted to α-keto acids by the branched-chain amino acid transaminase, followed by decarboxylation by the branched-chain α-keto acid dehydrogenase complex, resulting in branched-chain acyl-CoA intermediates. These intermediates are further converted to propionyl-CoA or acetyl-CoA through β-oxidation catalyzed by enzymes such as methylmalonic acid-semialdehyde dehydrogenase (*mmsA*), hydroxyisobutyrate dehydrogenase (*mmsB*), isovaleryl coenzyme A dehydrogenase (*ivd*), and enoyl-CoA hydratase (*paaF*). In the present study, the BCAA degradation pathway was not significantly altered in the mutant. However, the impairment of *rraA* resulted in decreases of 13.31- and 18.44-fold in MmsA (BAU10_20645 and BAU10_18450, respectively), 10.54-fold in MmsB (BAU10_20625), and 9.26-fold in PaaF (BAU10_18460 and BAU10_20670, respectively; Table S4), suggesting that β-oxidation of branched-chain acyl-CoA is also positively regulated by RraA (Fig. 6A and B).

In addition, several key enzymes related to amino acid metabolism were affected. Alanine dehydrogenase (Ald BAU10_04925) and glutamate dehydrogenase (Gdh BAU10_07330), which are responsible for the catabolism of alanine, aspartate, and glutamate and the corresponding carbon skeletons, were downregulated by 1.70–3.32-fold at the protein level. Interestingly, the expression of the large subunit of glutamate synthase (GltB BAU10_01395), which is involved in converting glutamine and 2-oxoglutarate into glutamate, increased 2.45-fold in the mutant (Table S4).

Furthermore, core enzymes in the arginine synthesis pathway, including argininosuccinate lyase ArgH (BAU10_13655), acetylglutamate kinase ArgB (BAU10_13665), N-acetyl-gamma-glutamyl-phosphate reductase ArgC (BAU10_13670), bifunctional N-succinyl diaminopimelate-aminotransferase or acetylornithine transaminase protein ArgD (BAU10_13850), ornithine carbamoyltransferase ArgF (BAU10_13125), and argininosuccinate synthase ArgG (BAU10_13660), were positively regulated by RraA (Fig. 6A and B; Tables S3 and S4).

Additionally, several genes encoding ABC transporters were upregulated (BAU10_06495 *ousX*, up 2.05-fold; BAU10_06505 *ousV*, up 2.39-fold; BAU10_09575 *oppF*, up 2.16-fold; BAU10_20535 *artP*, up 4.18-fold); and several ABC transporters were downregulated (BAU10_05985 *oppA*, down 3.37-fold; BAU10_18030, down 6.38-fold; BAU10_19275 *glnP* down 2.21-fold; BAU10_19280 *aapP* down 2.33-fold; BAU10_20520 *artM*, down 2.24-fold). However, in their protein levels, a large amount of the amino acid ABC transporters showed decreased trends in the *rraA* mutant, including MppA (BAU10_15390, down 2.52-fold), ArgT (BAU10_04000, down 3.42-fold), AapJ (BAU10_07410, down 3.37-fold), OusX (BAU10_06495, down 3.81-fold), OppA (BAU10_05985, down 24.48-fold), AotP (BAU10_03995, down 2.23-fold), OusV (BAU10_06505, down 3.29-fold), ArtP (BAU10_20535, down 1.78-fold), BAU10_18335(down 17.77-fold), SdaC (BAU10_20390, down 4.55-fold), OppA(BAU10_09280, down 1.86-fold), OppF (BAU10_05965, down 12.57-fold), and GlnQ (BAU10_07585, down 1.54-fold), suggesting that RraA may play a role in positively regulating the uptake of amino acids and maintaining the balance between carbon and nitrogen sources within the cell.

### RraA regulates genes involved in quorum sensing and the type VI secretion system

LuxQ-LuxU-LuxO is an important pathway for regulating biofilm formation in *Vibrio parahaemolyticus* (26). Deletion mutants of *luxQ*, *luxU*, and *luxO* formed weaker biofilms than did the WT strain. The data showed here that *luxN* (BAU10_09015) was downregulated by 2.26-fold at the mRNA level, while LuxO (BAU10_06930) was downregulated by 4.97-fold at the protein level, indicating that RraA may control biofilm formation by regulating the LuxQ-LuxU-LuxO pathway.

Bacterial secretion systems are macromolecular complexes that release virulence factors into the medium or translocate them into the target host cell (27). The type VI secretion system (T6SS) is widespread among pathogens and nonpathogens, and studies suggest that this system may be associated with interspecific competition and bacterial immunity to protect pathogens (28–30). Differentially expressed genes and proteins related to the T6SS were screened via transcriptomics and proteomics. Transcriptomic data revealed that T6SS-associated genes (*tssA* (BAU10_20250), *tssB1* (BAU10_20245), *tssC1* (BAU10_20240), *tssC2* (BAU10_20235), *tssG* (BAU10_20215), *BAU10_20275*, *tssM1* (BAU10_20265), *tssH* (BAU10_20210), and *vasD* (BAU10_20280) were significantly upregulated twofold to fourfold. In the *rraA* mutant strain, the expression of *tssM1* and *tssC1* was consistent with the transcriptome (Fig. 5). According to the proteomics data, only TagH was upregulated significantly, while the other DEGs exhibited no significant changes in protein levels. Mutations in the *rraA* gene activate the expression of most of the T6SS-related genes and several T6SS-related proteins, which suggests that *rraA* negatively and posttranscriptionally regulates the type VI secretion system.

## DISCUSSION

**Fig 7:**
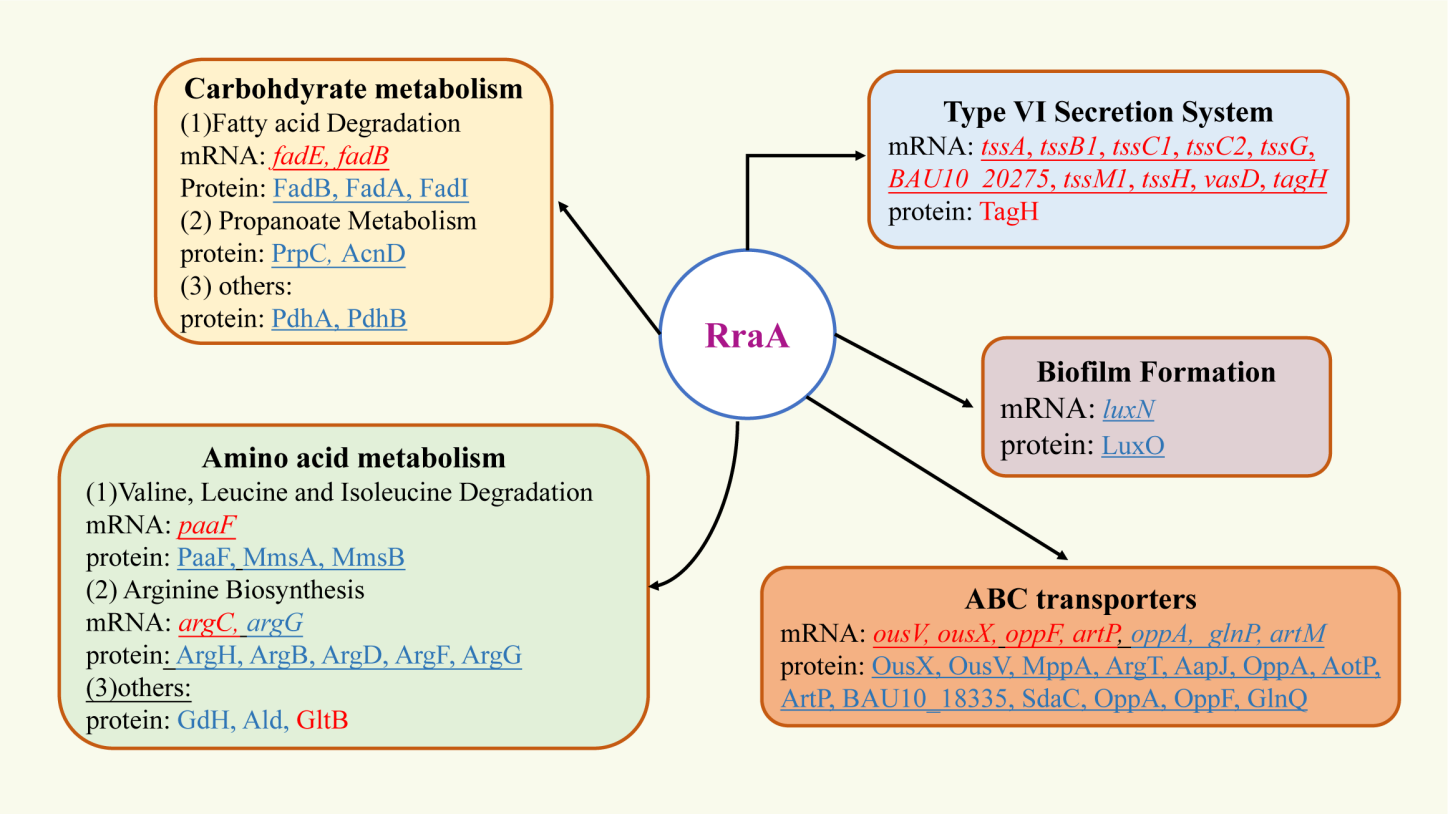
*V. alginolyticus* RraA regulon. Boxes indicate major pathways regulated by RraA. Solid arrows indicate those that have been confirmed by genetics. Genes or proteins in red are downregulated by *rraA* (upregulated in the *rraA* mutant), while genes or proteins in blue are upregulated by *rraA* (downregulated in the *rraA* mutant).

RraA has widespread effects on mRNA abundance and plays important physiological roles in growth and biofilm formation in *E. coli* and *V. vulnificus* (6, 7, 10). In this study, we characterized the physiological role of RraA in *V. alginolyticus* ZJ-T and identified its regulon, which is summarized in Fig. 7.

It was reported that the deletion of *rraA* resulted in reduced motility in *V. vulnificus* and *Salmonella* Typhimurium (12, 31). However, no significant impact on motility was observed for *V. alginolyticus*. Overexpression of RraA in *E. coli* was found to rescue cells from growth arrest, but this led to a decreased growth rate in *V. vulnificus* (12). These findings suggest that the function of RraA may have evolved to be species-specific.

Limited data exist on the regulation of *rraA*. An earlier study proposed that *rraA* in *E. coli* is transcribed from only one promoter, P*_rraA_*, located in the *menA-rraA* intergenic region, and the transcription of *rraA* increases during the stationary phase in a sigma(s)-dependent manner, as shown by primer extension and transcriptional fusion (4). Our study revealed the presence of at least three transcripts of different lengths, indicating the involvement of multiple promoters, including the conserved P*_rraA_* gene. Interestingly, in *V. alginolyticus*, the *rraA* mRNA level increased more than 12-fold during the stationary phase, but no detectable RraA protein was observed. This finding suggested the likelihood of posttranscriptional regulation, possibly through the inhibition of protein translation by an unknown factor. Similar expression patterns of the RraAV1 protein have been observed in *V. vulnificus*, indicating a potentially conserved mechanism.

This study utilized integrative transcriptome and proteome analysis to identify the regulon of *rraA* in *V. alginolyticus*. In comparison to the findings of a previous study conducted in *V. vulnificus*, the *rraA* regulon in *V. alginolyticus* exhibited significantly fewer transcriptional changes. This difference could be attributed to two factors. First, in a previous study, samples collected during the early stationary phase were used, while in our study, samples from the early log phase were used. Second, in a previous study, the criterion for differential gene expression was set at a 1.4-fold change, whereas we used a more stringent threshold of twofold.

Interestingly, only a few genes exhibited similar expression patterns for both DEGs and DEPs. This finding suggested that the regulation of *rraA* targets occurs primarily at the posttranscriptional level. For instance, in our study, the potential citrate catabolism operons *citCDEFXG* and *citS-oadGAB*, as well as the two-component system gene *citAB*, exhibited a significant decrease in transcript abundance (10–100-fold), but no difference in their protein products except for CitC was detected in the *rraA* mutant. One possible explanation is that RNase E is able to degrade both target mRNAs and small RNAs (sRNAs) that bind to target mRNAs and affect their translation. The impairment of *rraA* could lead to increased RNase E activity, resulting in decreased transcript abundance and the presence of unknown sRNAs that function as translational inhibitors. These opposing effects on protein synthesis could explain why the protein products of the affected genes showed no difference in the citrate metabolism operons. Notably, transcriptional, posttranscriptional, and translational regulation of citrate operons was reported in *Lactococcus lactis*, which further supports this hypothesis (32, 33).

Polymyxin B is a member of the colistin family of antibiotics that act by interacting with both the outer membrane and the inner membrane of bacteria (32). While resistance to this cationic lipopeptide is exceptionally rare (32), our findings indicate that disruption of the *rraA* gene leads to increased resistance to polymyxin B. Although the detailed mechanism remains unclear, previous reports have suggested a potential association between the reassembly of lipopolysaccharide and polymyxin B resistance in gram-negative bacteria (33, 34). In our investigation, we observed downregulation of the *lpxH* gene (BAU10_05120), which is known to be involved in lipid A biosynthesis and plays a crucial role in conferring polymyxin B resistance in gram-negative bacteria (33, 34). This finding suggested that *V. alginolyticus* RraA may facilitate the synthesis of lipid A, thereby conferring protection against polymyxin B-induced damage to cells.

Quorum sensing (QS) is a bacterial signaling pathway utilized to coordinate population behaviors, including biofilm formation, pathogenesis, and bioluminescence. In this study, we observed the downregulation of two QS proteins, LuxU (a phosphate transferase) and LuxO (a transcription factor activated by LuxU phosphorylation), at the protein level in the mutant. This finding suggested that RraA potentially has a positive impact on the QS signaling pathway by enhancing the translation of *luxU* and *luxO*. Previous reports have indicated that the deletion of *luxO* leads to reduced biofilm formation in *V. parahaemolyticus* and decreased expression of the cps biosynthesis gene cluster, which is responsible for producing major components of the biofilm matrix (26). Hence, it is reasonable to speculate that the decreased biofilm formation observed in the *rraA* mutant may be attributed to its positive influence on the LuxO and LuxU proteins.

Fatty acids serve as important carbon sources within cells when easily utilized carbon sources such as glucose are available. However, these compounds are degraded to support cell survival when these carbon sources become depleted. Fatty acids also play critical roles in maintaining membrane integrity and affecting various cellular processes (35, 36). According to the results of the proteomics analysis conducted in our study, *V. alginolyticus* RraA potentially positively regulates the expression of genes involved in the degradation of long-chain organic acids derived from fats or branched-chain amino acids through processes such as β-oxidation, the methylcitrate cycle, and branched-chain catabolism. These pathways collectively convert long-chain carbohydrates into important intermediates such as acetyl-CoA, propionyl-CoA, and pyruvate, which are required for energy production and metabolic remodeling.

In a previous study, the small RNA molecule FarS (fatty acid-regulated sRNA) in *V. cholerae*, produced through RNase E-mediated maturation of the *fadB* 3′-UTR, was reported to inhibit the expression of FadE, which is the rate-limiting enzyme involved in the oxidation of acyl-CoA (34). Based on this information, it is reasonable to speculate that *rraA* may derepress the expression of FadE by negatively affecting the production of a FarS homolog in *V. alginolyticus*.

In summary, our study identified RraA as a global regulator in *V. alginolyticus*. For the first time, we characterized its regulon at both the transcriptomic and proteomic levels, revealing complex interactions and mechanisms. However, further investigations are needed to determine the regulatory role of RraA in response to environmental cues and its involvement in posttranscriptional regulatory networks.

## Data Availability

All RNA-sequencing data are deposited in GenBank (wild-type biosample: SAMN29675353, SRA: SRR20124473-SRR20124475; *rraA* mutant strain biosample: SAMN30175538, SRA: SRR22012560-SRR22012562; accessed on 10 December 2022). All proteome data are deposited in iProX (Integrated Proteome Resources) database (https://www.iprox.cn/page/home. html, accessed on 22 September 2023); the accession ID is IPX0007150000. The other data presented in this study are available in the article.

## SUPPLEMENTAL MATERIAL

Supplemental material msphere.00020-24-s0001.pdf Figure S1 and Tables S1 to S3.

ASM does not own the copyrights to Supplemental Material that may be linked to, or accessed through, an article. The authors have granted ASM a non-exclusive, world-wide license to publish the Supplemental Material files. Please contact the corresponding author directly for reuse.
