# Supplementary material for: Identification of the global regulatory roles of RraA via the integrative transcriptome and proteome in Vibrio alginolyticus
Source: mSphere. 2024 Jun 27;9(7):e00020-24. doi: 10.1128/msphere.00020-24 (PMC11288022; doi:10.1128/msphere.00020-24)
Supplement: Supplemental material — Figure S1 and Tables S1 to S3. [file msphere.00020-24-s0001.pdf]

Figure S1 Amino acid sequence percent identity matrix of *rraA* in various bacteria

1: EcRraA	100.00	97.52	68.94	65.84	54.66	43.48	38.75	44.16	43.75	26.71	26.71
2: StRraA	97.52	100.00	68.94	65.84	54.04	43.48	37.50	44.16	43.12	26.09	26.09
3: VvRraAV1	68.94	68.94	100.00	86.90	57.41	39.52	41.88	42.21	48.78	24.86	24.86
4: VaRraA	65.84	65.84	86.90	100.00	58.02	39.51	41.25	41.56	43.83	23.81	23.81
5: PaRraA	54.66	54.04	57.41	58.02	100.00	48.77	46.25	43.51	42.24	29.01	29.01
6: VcRraA	43.48	43.48	39.52	39.51	48.77	100.00	76.69	45.51	39.26	21.84	21.84
7: VvRraAV2	38.75	37.50	41.88	41.25	46.25	76.69	100.00	42.68	40.88	22.35	22.02
8: MtRraA	44.16	44.16	42.21	41.56	43.51	45.51	42.68	100.00	45.75	31.85	31.41
9: TtRraA	43.75	43.12	48.78	43.83	42.24	39.26	40.88	45.75	100.00	32.93	32.93
10: ScRraA1	26.71	26.09	24.86	23.81	29.01	21.84	22.35	31.85	32.93	100.00	100.00
11: ScRraA2	26.71	26.09	24.86	23.81	29.01	21.84	22.02	31.41	32.93	100.00	100.00

Table S1 Antibiotics resistance of ZJ-T, ZJ-T-Δ*rraA* and ZJ-T/over *rraA*-pSCT32

Antibiotic	Concentration （ug.piece^{-1}）	Size of inhibition zone/mm		
		ZJ-T	ZJ-T-Δ*rraA*	ZJ-T/over *rraA*-pSCT32
Clindamycin	2	0.00/R	0.00/R	0.00/R
Chloroamphenicol	30	24.67±0.58/S	24.57±2.08/S	24.87±1.14/S
Furazolidone	300	15.00±1.00/I	16.83±1.04/I	14.60±2.25/I
Compound sulfamethoxazoles	1.25	0.00/R	0.00/R	0.00/R
Polymyxin B	300 IU	**15.00±1.00/I**	**0.00/R**	**0.00/R**
Vancomycin	30	0.00/R	0.00/R	0.00/R
Ciprofloxacin	5	13.17±3.69/R	13.80±1.06/R	12.67±1.89/R
Oflaoxacin	5	13.17±2.02/I	13.47±3.22/I	13.30±1.81/I
norfloxacin	10	12.90±1.10/I	12.00±1.00/I	12.10±1.80/I
Midecamycinum	30	11.60±1.78/R	119.60±1.44/R	10.67±6.81/R
Erythromycin	15	15.27±3.20/I	14.33±1.53/I	12.33±3.23/I
Minocycline	30	18.93±4.31/S	19.90±4.25/S	18.13±7.94/S
Doxycycline	30	17.40±1.97/I	17.67±1.15/I	16.67±1.45/I
Tetracycline	30	17.33±1.15/I	17.67±0.58/I	16.27±0.46/I
Neomycin	30	14.07±0.90/I	16.90±0.14/I	14.60±1.64/I
kanamycin	30	13.57±1.89/I	12.53±0.76/I	12.80±1.31/I
Gentamicin	10	13.27±1.55/I	12.33±1.10/I	11.73±2.61/I
Amikacin	30	12.07±1.79/R	14.33±1.53/R	13.73±0.71/R

Cefoperazone	75	0.00/R	0.00/R	0.00/R
Ceftazidime	30	16.70±3.50/I	16.70±2.10/I	17.20±2.80/I
Cefuroxim	30	9.70±0.60/R	12.0±2.60/R	12.00±1.00/R
Cefradine	30	0.00±0.00/R	6.00±5.30/R	0.00±0.00/R
Cefamezin	30	8.00±7.00/R	9.00±7.80/R	12.90±1.20/R
Cephalexin	30	4.00±6.90/R	11.30±4.00/R	10.30±1.20/R
Piperacillin	100	0.00±0.00/R	0.00±0.00/R	0.00±0.00/R
Carbenicillin	100	0.00/R	0.00/R	0.00/R
Ampicillin	10	0.00/R	0.00/R	0.00/R
Oxacillin	1	0.00/R	0.00/R	0.00/R
Penicillin	10 IU	0.00/R	0.00/R	0.00/R

Note: S. Susceptible; I. Intermediate; R. Resistance

Table S2 Nucleotide sequences of primers used in this study

Primers	Sequence (5’-3’)
For *rraA* mutant strain construction	
pSW7848-F	GTCTGATTCGTTACCAATTATGACAAC
pSW7848-R	GAATTCGATATCAAGCTTATCGATAC
RraA-UP-F	ataagcttgatatcgaattcAGCGGATTTTATATTGTTAACAG
RraA-UP-R	agtccagcttTGTAATGTCTCTCATATTTCC
RraA-DOWN-F	agacattacaAAGCTGGACTTCTAAAAAC
RraA-DOWN-R	taattggtaacgaatcagacATGTTCATAGGATCGAATTATTTAG
Del-check-pSW7848-F	TCACTGTCCCTTATTCGCACC
Del-check-pSW7848-R	CTGCTTTTGAGCACTACCCG
Δ-RraA-check-F	CTCCATTAAGCCTCGGCTCG
Δ-RraA-chenk-R	ACCAAGATTGGCAACAAGGC
For *rraA* complementary strain construction	
pMMB207-F	AGAAGCGGTCTGATAAAACAGAATTTGC
pMMB207-R	GCGCAACGCAATTAATGTAAGTTAG
tac-com-RraA-F	tataatgtgtggaattgtgaCGCTTGGGTGAGAAAATTC
tac-com-RraA-R	ctgatttaatctgtatcaggCGCGACTCTTGATTACCAAG
pMMB207-check-F	TCTCTTCCGGGCGCTATCATG
pMMB207-check-R	TCGTTTTATTTGATGCCTGGCAG
For *rraA* overexpression strain construction	
over-RraA-F	TGTGACCAATTTGAAGACCAAGT
over-RraA-R	TGAGGTTGGTTTAGGCTTTCGT
over-pSCT32-F	ATAAGCTTGATATCGAATTCTGCACGCTCGCTTACCGG
over-pSCT32-R	AAATTAGCATTCTTTGCTCCTTGAGTAATCTCTAAATTTTCTT GC
For 5'-RACE	
5' adaptor Primer	GCTGTCAACGATACGCTACGTAACGGCATGACAGTGGGGGG GGGGGGG
5'-RACE Outer Primer	GCTGTCAACGATACGCTACGTAAC
5'-RACE-*rraA*-R1	GGTAGCCGTTGAATTGGCG
5'-RACE-*rraA*-R2	ACTGGGCGAAGATGGAACG
5'-RACE-*rraA*-R3	TGGGTGGGAAGGCATCATTG
5'-RACE-*rraA*-R4	ACTACGTTTACGCGGACTGG
For translational fusions	
pSCT32-gfp_F	GTGAGCAAGGGCGAGGAGCT
pSCT32-gfp_R	TAAATGCATGCCGCTTCGCCTT
pSCT32-gfp-check-F	CGCTATCGCTACGTGACTGG
pSCT32-gfp-check-R	GTTTCACTTCTGAGTTCGGCATG
rraA-TL-F	ggcgaagcggcatgcatttaAGGAACATTAAAGCAATGTTC
rraA-TL-R	agctcctcgcccttgctcacAATATCGGGTGTAATGTC
For qRT-PCR	

luxO-F	CAGAGCGACCGTAAAGACAAG
luxO-R	CTGTTGTTGCTCCAGTAAAGGC
csgD-F	GAAGAACTCGCTTCTTGGCCT
csgD-R	TCAAGTAATCTTCGAGACAGCCA
tssC1-F	TTCACCAGCACTATTCGGCTT
tssC1-R	GCACTCTCGCGGAACGAAC
tssM1-F	CACTAGAATACGCCCAATGGC
tssM1-R	ACTCTTCGACTGGAGCGGTA
etk-F	AAATCTTCGCAAAGTTTAGACAGC
etk-R	GTAGCGCTGTGCCAGTTCA
BAU10_21865-F	GCGCAATTGCATAAGCCTCA
BAU10_21865-R	TTTCGTCCGATCGGTTCTCG

Table S3 *Vibrio alginolyticus* genes are up/down regulated significantly in *rraA* mutant.
Orange shade: Differentially expressed genes mentioned in the article.

id	Symbol	Description	TR-FC	FDR
BAU10_00035	--	oligosaccharide flippase family protein	-2.2860	0.0009
BAU10_00040	--	glycosyltransferase family 2 protein	-2.1894	0.0061
BAU10_00145	--	site-specific integrase	-2.2383	0.0000
BAU10_00175	--	helix-turn-helix transcriptional regulator	2.0907	0.0000
BAU10_00190	--	hypothetical protein	-2.8149	0.0015
BAU10_00280	*hslU*	heat shock protein HslVU, ATPase subunit HslU	2.3158	0.0000
BAU10_00285	*hslV*	ATP-dependent protease HslV	2.0877	0.0000
BAU10_00335	*rpsS*	30S ribosomal protein S9	2.1475	0.0000
BAU10_00340	*rplV*	50S ribosomal protein L22	2.0133	0.0000
BAU10_00355	*rpmC*	50S ribosomal protein L29	2.2501	0.0000
BAU10_00485	*cysG*	uroporphyrinogen-III C-methyltransferase	2.1121	0.0000
BAU10_00490	*cysD*	sulfate adenylyltransferase subunit 2	2.2986	0.0000
BAU10_00800	*glmS*	glucosamine--fructose-6-phosphate aminotransferase (isomerizing)	-2.2784	0.0000
BAU10_00850	*mtlD*	mannitol--phosphate 5-dehydrogenase	-2.9582	0.0000
BAU10_01015	*yqeY*	Transamidase GatB domain protein	-2.6599	0.0000
BAU10_01210	*diaA*	putative phosphoheptose isomerase	-2.1743	0.0000
BAU10_01335	--	hypothetical protein	-2.4414	0.0020
BAU10_01360	*btuF*	vitamin B2 ABC transporter substrate-binding protein BtuF	-2.7369	0.0000
BAU10_01365	*cobD*	cobalamin biosynthesis family protein	-2.5955	0.0000
BAU10_01400	*yhcC*	TIGR022 family radical SAM protein	-2.2515	0.0000
BAU10_01430	*RraA*	ribonuclease E inhibitor RraA	-246.1481	0.0000
BAU10_01460	*grcA*	formate acetyl transferase-related protein	3.1420	0.0000
BAU10_01540	*zraR*	sigma-54 dependent transcriptional regulator	2.0687	0.0000
BAU10_01605	*hlyU*	transcriptional activator HlyU	-2.1899	0.0000
BAU10_01660	--	putative carbon starvation protein A	-2.1269	0.0000
BAU10_01735	*yfiA*	putative sigma-54 modulation protein	2.2467	0.0000
BAU10_01760	*clpB*	ATP-dependent chaperone ClpB	2.0636	0.0000
BAU10_02360	--	DUF3622 domain-containing protein	-2.4970	0.0019
BAU10_02380	--	DUF3332 family protein	2.1651	0.0019
BAU10_02545	*metQ*	lipoprotein YaeC	2.1840	0.0000
BAU10_02810	*lolB*	lipoprotein localization protein LolB	-2.1803	0.0000
BAU10_02895	--	copper amine oxidase	2.0011	0.0013
BAU10_02900	*copA*	copper-translocating P-type ATPase	10.4461	0.0000
BAU10_02920	--	hypothetical protein	3.2224	0.0000
BAU10_02925	*ompA1*	outer membrane protein OmpA	2.6443	0.0000
BAU10_03050	*flaE*	flagellin	-2.0206	0.0000
BAU10_03145	--	selenoprotein W-related protein	-2.5169	0.0028
BAU10_03150	--	sulfite exporter TauE/SafE family protein	-4.0674	0.0000
BAU10_03165	*htpG*	molecular chaperone HtpG	2.1408	0.0000
BAU10_03345	--	membrane protein	-2.5620	0.0266
BAU10_03535	--	putative phospholipid biosynthesis acyltransferase	-3.3257	0.0000
BAU10_03550	--	methyltransferase domain-containing protein	-2.0314	0.0000
BAU10_03655	--	rhombosortase	-3.0953	0.0000
BAU10_03715	*VP0936*	putative sulfate exporter family transporter	-2.8353	0.0000
BAU10_03720	*lplT*	acyl-phosphate glycerol 3-phosphate acyltransferase	-3.1946	0.0000

BAU10_04030	*priC*	primosomal replication protein N"	-2.1696	0.0000
BAU10_04080	*infA*	Translation initiation factor IF-	-2.5142	0.0000
BAU10_04125	--	alpha/beta hydrolase	-2.1278	0.0000
BAU10_04160	*elyC*	envelope biogenesis factor ElyC	-2.0233	0.0000
BAU10_04220	*PBPRA3*	YebC/PmpR family DNA-binding transcriptional regulator	2.2416	0.0000
BAU10_04330	*alaE*	L-alanine exporter AlaE	-2.3245	0.0000
BAU10_04360	--	MULTISPECIES: hypothetical protein	-14.1463	0.0115
BAU10_04425	--	phosphohydrolase	-2.2934	0.0047
BAU10_04450	--	HigA family addiction module antidote protein	2.1581	0.0039
BAU10_04500	--	type IV secretion system protein TraC	2.2143	0.0253
BAU10_04605	*ssb*	Single-stranded DNA-binding protein	1493.3333	0.0152
BAU10_04700	--	hypothetical protein VH709_contig00050-027	-81.7995	0.0000
BAU10_04705	*dpiA*	response regulator	-2.9674	0.0000
BAU10_04710	*citA*	sensor histidine kinase	-9.1137	0.0000
BAU10_04715	*oadB*	oxaloacetate decarboxylase, beta subunit	-13.5250	0.0000
BAU10_04720	*oadA*	oxaloacetate decarboxylase alpha subunit	-17.6992	0.0000
BAU10_04725	*oadG2*	oxaloacetate decarboxylase	-30.8077	0.0000
BAU10_04730	*citP*	Na(+)citrate OH(-) antiporter	-32.5623	0.0000
BAU10_04735	*citC*	citrate carrier	-354.3796	0.0000
BAU10_04740	*citD*	citrate lyase acyl carrier protein	-21.8218	0.0000
BAU10_04745	*citE*	citrate (pro-3S)-lyase subunit beta	-18.9873	0.0000
BAU10_04750	*citF*	citrate lyase subunit alpha	-15.1257	0.0000
BAU10_04755	*citX*	holo-ACP synthase CitX	-165.1630	0.0000
BAU10_04760	*citG*	2-(5''-triphosphoribosyl)-3'-dephospho-CoA synthase	-163.9326	0.0000
BAU10_04765	--	hypothetical protein	-95.1028	0.0000
BAU10_05015	--	zinc ABC transporter permease	-2.8621	0.0147
BAU10_05045	--	hypothetical protein	-2.6798	0.0107
BAU10_05120	*lpxH*	UDP-2,3-diacylglucosamine diphosphatase	-2.7719	0.0000
BAU10_05300	*HI_0042*	TIGR062 family pseudouridine synthase	-2.7190	0.0001
BAU10_05450	--	transcriptional regulator, TetR family	4.8065	0.0000
BAU10_05495	*lysO*	membrane protein	-2.0966	0.0000
BAU10_05505	*tfoX*	DNA transformation protein	-2.5778	0.0000
BAU10_05680	--	sporulation protein	2.0350	0.0000
BAU10_05790	*VP300*	antiholin-like protein LrgA	-2.7338	0.0000
BAU10_05845	*btuD*	vitamin B2 ABC transporter ATP-binding protein BtuD	-2.0561	0.0001
BAU10_05985	*oppA*	peptide ABC transporter substrate-binding protein	-3.3725	0.0173
BAU10_05990	*yqjE*	M20/M25/M40 family metallo-hydrolase	-2.1129	0.0000
BAU10_06130	*CPA*	N-carbamoylputrescine amidase	2.3101	0.0219
BAU10_06160	*pecS*	transcriptional regulator, MarR family protein	2.2008	0.0000
BAU10_06370	*xseA*	exodeoxyribonuclease VII large subunit	2.8176	0.0000
BAU10_06390	*N4bp2l*	AAA family ATPase	2.5111	0.0140
BAU10_06495	*ousX*	L-proline glycine betaine binding ABC transporter protein ProX	2.0481	0.0000
BAU10_06505	*ousV*	ABC superfamily (glycine/betaine/proline transport protein)	2.3856	0.0000
BAU10_06530	*ectB*	Diaminobutyrate--2-oxoglutarate transaminase	2.2615	0.0000
BAU10_06535	*ectC*	ectoine synthase	2.2779	0.0007
BAU10_06675	*rhtB*	putative homoserine/homoserine lactone efflux protein	-2.1292	0.0041
BAU10_06680	*yjjI*	YjjI family glycine radical enzyme	-2.0204	0.0000

BAU10_06685	*yjjW*	YjjW family glycine radical enzyme activase	-2.3490	0.0000
BAU10_06935	--	Hpt domain-containing protein	6.9962	0.0000
BAU10_06995	*dgcC*	diguanylate cyclase	-2.2260	0.0000
BAU10_07295	*rmf*	ribosome modulation factor	2.0377	0.0000
BAU10_07450	*acyP*	acylphosphate phosphohydrolase	3.7176	0.0357
BAU10_07550	*CRYZ*	zinc-binding dehydrogenase	2.0646	0.0001
BAU10_07700	*prpB*	carboxyphosphonoenolpyruvate phosphonomutase	3.4957	0.0000
BAU10_07910	*yscG*	putative type III secretion protein	2.7632	0.0010
BAU10_07915	*pscF*	type III export protein YscF	2.9519	0.0015
BAU10_08025	--	Cation transport ATPase	2.1360	0.0053
BAU10_08260	--	damage-inducible protein J	2.0298	0.0001
BAU10_08505	--	hypothetical protein	5.0189	0.0033
BAU10_08620	--	EAL domain-containing protein	-4.0292	0.0000
BAU10_08670	--	porin family protein	-2.2683	0.0000
BAU10_08755	--	imelysin family protein	2.5801	0.0001
BAU10_08815	--	hypothetical protein	-2.1100	0.0000
BAU10_08820	--	tetratricopeptide repeat protein	-2.1708	0.0000
BAU10_08865	*nspC*	carboxynorspermidine decarboxylase	3.1475	0.0000
BAU10_08870	*N646_023*	Carboxynorspermidine synthase	2.8272	0.0000
BAU10_08900	*pgsA*	CDP-diacylglycerol--glycerol-3-phosphate 3-phosphatidyltransferase	-2.0053	0.0000
BAU10_09015	*luxN*	hybrid sensor histidine kinase/response regulator	-2.2644	0.0000
BAU10_09240	*ttrA*	tetrathionate reductase subunit TtrA	2.0475	0.0000
BAU10_09255	*pqiA*	paraquat-inducible protein A	-2.1961	0.0000
BAU10_09525	*dgcT*	putative GGDEF family protein	-2.6647	0.0000
BAU10_09575	*oppF*	oligopeptide ABC transporter, ATP-binding protein	2.1586	0.0000
BAU10_09610	*moaC*	molybdenum cofactor biosynthesis protein C	-2.2901	0.0000
BAU10_09620	*moaA*	GTP 3',8-cyclase MoaA	-2.0752	0.0000
BAU10_09700	*motY*	sodium-type flagellar protein MotY	-2.9465	0.0000
BAU10_09905	*gloA*	putative lactoylglutathione lyase	-2.1168	0.0005
BAU10_09910	--	antibiotic biosynthesis monooxygenase	-2.1184	0.0008
BAU10_09930	*aqpZ*	aquaporin Z	3.6471	0.0000
BAU10_10155	*ccmB*	heme exporter protein B	-3.0025	0.0000
BAU10_10225	*flhB*	flagellar biosynthesis protein B	-2.7314	0.0000
BAU10_10230	*fliR*	flagellar biosynthetic protein FliR	-2.7926	0.0000
BAU10_10235	*fliQ*	flagellar biosynthesis protein Q	-3.5599	0.0000
BAU10_10240	*fliP*	flagellar type III secretion system pore protein FliP	-2.8032	0.0000
BAU10_10275	--	flagellar biosynthesis chaperone	-3.2047	0.0000
BAU10_10375	--	conserved hypothetical protein	-2.3385	0.0000
BAU10_10465	*purM*	Phosphoribosylformylglycinamidine cyclo-ligase	-2.5262	0.0004
BAU10_10485	*fadE*	acyl-CoA dehydrogenase	2.1117	0.0007
BAU10_10620	*cdsA*	Phosphatidate cytidylyltransferase	-2.3189	0.0000
BAU10_10625	*uppS*	undecaprenyl diphosphate synthase	-2.3229	0.0000
BAU10_10665	*truC*	tRNA pseudouridine synthase C	-2.2817	0.0020
BAU10_10685	--	GNAT family N-acetyltransferase	-3.6762	0.0000
BAU10_10700	--	ketoacyl-ACP synthase III	-2.6098	0.0303
BAU10_10725	--	hypothetical protein	-2.8340	0.0000
BAU10_10795	--	DUF2789 domain-containing protein	2.4084	0.0002

BAU10_10940	--	hypothetical protein	-2.5848	0.0000
BAU10_10990	--	nitrogen-fixing protein NifU	2.1321	0.0002
BAU10_10995	--	GGGtGRT protein	2.1827	0.0006
BAU10_11075	--	dentin sialophosphoprotein	2.7203	0.0138
BAU10_11120	--	PilZ domain-containing protein	-2.0432	0.0000
BAU10_11155	*HI_059*	NupC family protein	-2.0348	0.0000
BAU10_11250	*pleD*	GGDEF domain protein	-2.0237	0.0001
BAU10_11430	--	conjugative transfer protein TraI	-2.4160	0.0006
BAU10_11505	*str*	streptomycin resistance protein B	-2.0544	0.0000
BAU10_11595	--	hypothetical protein	4.3060	0.0000
BAU10_11625	*ubiV*	U32 family peptidase	-2.2153	0.0000
BAU10_11925	--	efflux transporter periplasmic adaptor subunit	-2.3086	0.0000
BAU10_11950	*evgS*	response regulator	-2.0397	0.0000
BAU10_12030	*VP2494*	YacL family protein	1666.6667	0.0181
BAU10_12135	*aceF*	pyruvate dehydrogenase complex dihydrolipoyllysine-residue acetyltransferase	2.0026	0.0000
BAU10_12140	*aceE*	pyruvate dehydrogenase (acetyl-transferring), homodimeric type	2.0703	0.0000
BAU10_12220	*ypjD*	inner membrane protein YpjD	-3.1150	0.0000
BAU10_12535	*ygfA*	5-formyltetrahydrofolate cyclo-ligase	-2.6107	0.0000
BAU10_12615	*sprT*	SprT protein	2.3075	0.0024
BAU10_13100	--	DUF206 domain-containing protein	-3.2425	0.0000
BAU10_13275	*ylaK*	PhoH family protein	2.0623	0.0003
BAU10_13300	*VP2688*	Maf-like protein	-2.1893	0.0000
BAU10_13310	*mreC*	rod shape-determining protein MreC	-2.4252	0.0000
BAU10_13495	*cysI*	assimilatory sulfite reductase (NADPH) hemoprotein subunit	2.1409	0.0000
BAU10_13500	*cysJ*	sulfite reductase	2.6846	0.0000
BAU10_13660	*argG*	argininosuccinate synthase	-2.4271	0.0000
BAU10_13670	*argC*	N-acetyl-gamma-glutamyl-phosphate reductase	2.0888	0.0012
BAU10_13685	--	predicted transcriptional regulator	-2.3163	0.0000
BAU10_13720	*bfd*	bacterioferritin-associated ferredoxin	2.9305	0.0129
BAU10_13745	*tusB*	sulfurtransferase complex subunit TusB	-2.3826	0.0000
BAU10_13750	*tusC*	sulfurtransferase complex subunit TusC	-2.0300	0.0000
BAU10_13835	--	DUF338 domain-containing protein	2.0585	0.0000
BAU10_13860	--	MarR family transcriptional regulator	2.4856	0.0000
BAU10_13865	*pecM*	EamA family transporter	3.5000	0.0000
BAU10_14050	--	DMT family transporter	-2.0305	0.0001
BAU10_14110	*frdB*	succinate dehydrogenase	-2.1093	0.0000
BAU10_14115	*frdC*	Fumarate reductase subunit C	-2.1079	0.0000
BAU10_14120	*frdD*	fumarate reductase subunit D	-2.0550	0.0000
BAU10_14475	*yhgN*	multiple antibiotic resistance protein marC	-3.2043	0.0000
BAU10_14490	--	DUF445 domain-containing protein	2.2714	0.0000
BAU10_14515	*gap3*	glyceraldehyde 3-phosphate dehydrogenase	2.0200	0.0000
BAU10_14700	*yhhQ*	putative preQ0 transporter	3.1877	0.0011
BAU10_14765	*mdtL*	multidrug resistance protein, putative	-2.0354	0.0000
BAU10_15010	*tnpR*	resolvase	2.0372	0.0043
BAU10_15015	--	hypothetical protein	3.5507	0.0000
BAU10_15210	*ibpA*	6 kDa heat shock protein A	2.9344	0.0000

BAU10_15270	*fadB*	fatty acid oxidation complex subunit alpha FadB	2.3810	0.0002
BAU10_15450	*mdtI*	multidrug resistance protein MdtI	3.7166	0.0035
BAU10_15480	*xapA*	xanthosine phosphorylase	-7.7074	0.0000
BAU10_15525	*bioP*	DMT family transporter	-2.6426	0.0000
BAU10_15615	--	EamA family transporter	-2.1083	0.0000
BAU10_15710	*PA727*	EAL domain-containing protein	-2.3138	0.0000
BAU10_15810	*epsH*	prepilin-type N-terminal cleavage/methylation domain-containing protein	-2.3768	0.0000
BAU10_16535	--	hypothetical protein	-2.1187	0.0183
BAU10_16540	--	C factor cell-cell signaling protein	2.1772	0.0136
BAU10_16630	*ulaA*	ascorbate-specific PTS system enzyme IIC	-14.7107	0.0000
BAU10_16635	--	PTS sugar transporter subunit IIB	-17.7375	0.0000
BAU10_16640	*ulaC*	PTS mannitol transporter subunit IIA	-5.6459	0.0000
BAU10_16715	*ompA*	OmpA family protein	2.9149	0.0000
BAU10_16730	--	hypothetical protein	2.2388	0.0106
BAU10_16760	*mltD*	lytic transglycosylase domain-containing protein	-4.1235	0.0003
BAU10_16955	--	Nudix-related transcriptional regulator NrtR	2.7841	0.0000
BAU10_16960	*pncB*	nicotinate phosphoribosyltransferase	2.8745	0.0000
BAU10_16990	--	putative outer membrane protein	-3.4370	0.0000
BAU10_17140	--	TetR/AcrR family transcriptional regulator	2.7032	0.0003
BAU10_17190	--	hypothetical protein	-2.1521	0.0008
BAU10_17235	*purA2*	adenylosuccinate synthase	2.7685	0.0000
BAU10_17310	*nemA*	alkene reductase	4.3237	0.0003
BAU10_17425	*scrY*	carbohydrate porin	-2.0612	0.0001
BAU10_17460	--	putative DamX-related protein	-2.7208	0.0420
BAU10_17475	--	adenosine deaminase	-5.3615	0.0044
BAU10_17480	--	hypothetical protein	-6.5696	0.0001
BAU10_17485	*cspA*	cold shock transcriptional regulator CspA	-17.3939	0.0000
BAU10_17520	*dgkA*	diacylglycerol kinase	-2.8645	0.0094
BAU10_17525	--	hypothetical protein	-2.1594	0.0079
BAU10_17530	*rstB*	sensor histidine kinase	-2.1775	0.0000
BAU10_17770	--	ATPase	-2.1179	0.0027
BAU10_17870	*pepT*	peptidase T	-2.3575	0.0000
BAU10_17890	*hlyB*	HAMP domain-containing protein	-2.4583	0.0000
BAU10_17990	--	hypothetical protein	-2.0910	0.0000
BAU10_18030	--	ABC transporter substrate-binding protein	-6.3816	0.0014
BAU10_18220	*RF_0358*	multidrug effflux MFS transporter	2.4435	0.0016
BAU10_18285	*pagL*	6-phospho-alpha-glucosidase	7.2669	0.0000
BAU10_18355	*fabG*	SDR family oxidoreductase	2.7471	0.0000
BAU10_18445	*thlA*	thiolase family protein	3.2941	0.0001
BAU10_18455	*ACAD8*	acyl-CoA dehydrogenase	4.2746	0.0011
BAU10_18490	*betB*	betaine-aldehyde dehydrogenase	2.7062	0.0000
BAU10_19230	*fruA*	PTS fructose transporter subunit IIBC	2.7956	0.0000
BAU10_19235	*fruK*	1-phosphofructokinase	6.8853	0.0000
BAU10_19240	*fruB*	bifunctional PTS fructose transporter subunit IIA/HPr protein	5.9005	0.0008
BAU10_19260	*AZC_3085*	diguanylate phosphodiesterase	-2.3485	0.0000
BAU10_19275	*glnP*	polar amino acid ABC transporter permease	-2.2128	0.0001

BAU10_19280	*aapP*	amino acid ABC transporter ATP-binding protein	-2.3330	0.0000
BAU10_19375	*focA*	formate transporter FocA	-2.1716	0.0000
BAU10_19405	--	hypothetical protein	2.5170	0.0000
BAU10_19580	--	hypothetical protein	3.6639	0.0000
BAU10_19585	--	DUF998 domain-containing protein	4.0423	0.0000
BAU10_19590	--	DUF2075 domain-containing protein	4.5927	0.0000
BAU10_19595	--	hypothetical protein	4.5301	0.0000
BAU10_19600	--	AAA family ATPase	3.3549	0.0000
BAU10_19605	*yqhH*	DEAD/DEAH box helicase	3.3896	0.0000
BAU10_19610	*MJECS02*	hypothetical protein	3.1174	0.0000
BAU10_19615	*yqhH*	DEAD/DEAH box helicase family protein	2.9192	0.0000
BAU10_19620	--	hypothetical protein	2.6092	0.0000
BAU10_19625	*MJ59*	AAA family ATPase	3.6075	0.0000
BAU10_19630	--	DEAD/DEAH box helicase	3.4484	0.0000
BAU10_19635	*rep*	DNA helicase	3.6662	0.0000
BAU10_19670	*Bmul_4720*	transposase	6.7053	0.0277
BAU10_19675	--	ATP-binding protein	2.9118	0.0000
BAU10_19680	*yrrC*	AAA family ATPase	3.2625	0.0000
BAU10_19685	--	hypothetical protein	3.3528	0.0002
BAU10_19690	--	hypothetical protein	3.0723	0.0000
BAU10_19695	--	hypothetical protein	2.6564	0.0000
BAU10_19700	--	site-specific integrase	2.4146	0.0000
BAU10_19705	--	site-specific integrase	2.2238	0.0000
BAU10_19770	*pleD*	GGDEF domain-containing protein	-2.6099	0.0000
BAU10_19785	*modC*	molybdenum ABC transporter ATP-binding protein ModC	-2.3471	0.0000
BAU10_19790	*modB*	molybdate ABC transporter permease subunit	-2.1013	0.0000
BAU10_19890	*ydeP*	FdhF/YdeP family oxidoreductase	2.7386	0.0000
BAU10_19900	*fdhD*	formate dehydrogenase family accessory protein FdhD	2.8070	0.0000
BAU10_19910	*uhpA*	transcriptional regulator UhpA	-2.8231	0.0021
BAU10_19915	*uhpB*	signal transduction histidine-protein kinase/phosphatase UhpB	-2.7180	0.0000
BAU10_20115	*dld*	D-lactate dehydrogenase	2.8869	0.0000
BAU10_20155	--	cytochrome c553	7.7266	0.0000
BAU10_20205	--	Putative cytoplasmic protein USSDB7A	2.2930	0.0000
BAU10_20210	*tssH*	type VI secretion system ATPase TssH	3.6085	0.0000
BAU10_20215	*tssG*	type VI secretion system baseplate subunit TssG	2.9399	0.0000
BAU10_20220	*tssF*	VAT2t	2.9010	0.0000
BAU10_20225	--	lysozyme	2.3397	0.0009
BAU10_20230	--	protein of avirulence locus	4.3501	0.0000
BAU10_20235	*tssC2*	type VI secretion system contractile sheath large subunit	2.5604	0.0000
BAU10_20240	*tssC1*	type VI secretion system contractile sheath large subunit	4.2778	0.0000
BAU10_20245	*tssB1*	type VI secretion protein	3.6842	0.0000
BAU10_20250	*tssA*	type VI secretion system protein TssA	2.6115	0.0000
BAU10_20255	*prpC*	phosphoprotein phosphatase	2.2260	0.0019
BAU10_20265	*tssM1*	type VI secretion system membrane subunit TssM	2.9486	0.0000
BAU10_20270	*tssL*	type VI secretion system protein TssL	3.3151	0.0000
BAU10_20275	--	type VI secretion system-associated protein	2.6661	0.0000
BAU10_20280	*vasD*	type VI secretion lipoprotein/VasD	2.8214	0.0000

BAU10_20285	*tagH*	type VI secretion system-associated FHA domain protein TagH	2.9842	0.0000
BAU10_20290	*prkC*	serine/threonine protein kinase	3.1916	0.0000
BAU10_20400	--	hypothetical protein	-3.6674	0.0000
BAU10_20435	--	hypothetical protein	3.6934	0.0495
BAU10_20445	--	hypothetical protein	2.2344	0.0002
BAU10_20450	*RP573*	copper resistance protein CopZ	3.3580	0.0000
BAU10_20490	*PA2953*	electron transfer flavoprotein-ubiquinone oxidoreductase	2.0960	0.0359
BAU10_20520	*artM*	arginine ABC transporter permease ArtM	-2.2434	0.0052
BAU10_20535	*artP*	arginine ABC transporter ATP-binding protein ArtP	4.1781	0.0001
BAU10_20575	*gucD*	aldehyde dehydrogenase family protein	3.2429	0.0093
BAU10_20590	*Dred_242*	NADH:flavin oxidoreductase	-2.7967	0.0000
BAU10_20655	*hmrR*	putative transcriptional regulator	4.0683	0.0494
BAU10_20670	*paaF*	gamma-carboxygeranoyl-CoA hydratase	2.0512	0.0050
BAU10_20710	*yciC*	cobalamin biosynthesis protein CobW	0.4935	0.0340
BAU10_20875	--	hypothetical protein	2.4797	0.0000
BAU10_20890	*cspA*	cold shock transcriptional regulator CspA	-6.7814	0.0000
BAU10_20935	*ypeB*	Putative cytoplasmic protein	-3.2725	0.0340
BAU10_20980	--	cation diffusion facilitator family transporter	-2.7523	0.0000
BAU10_21015	*HI_349*	DPS family protein	2.5533	0.0000
BAU10_21045	*mak2*	response regulator	-3.0482	0.0003
BAU10_21050	*tolC*	TolC family protein	-2.4639	0.0014
BAU10_21060	*abcE*	ATP-binding cassette domain-containing protein	-2.3864	0.0011
BAU10_21080	--	Alternative ribosome-rescue factor A	2.1225	0.0119
BAU10_21085	*cspE*	Cold shock protein CspE	-2.2117	0.0000
BAU10_21090	--	YaeQ protein	-2.7832	0.0000
BAU10_21145	*MT-CO3*	cytochrome c oxidase, subunit III	-3.6509	0.0079
BAU10_21250	--	GFA family protein	2.9640	0.0465
BAU10_21330	--	hypothetical protein	2.3173	0.0001
BAU10_21385	--	hypothetical protein	4.0032	0.0000
BAU10_21510	--	hypothetical protein	-2.4733	0.0008
BAU10_21680	*exbB*	TonB system transport protein ExbB	16.5161	0.0007
BAU10_21720	--	hypothetical protein	-2.7792	0.0000
BAU10_21845	*wcaJ*	undecaprenyl-phosphate glucose phosphotransferase	3.4286	0.0021
BAU10_21860	*etk*	polysaccharide biosynthesis tyrosine autokinase	2.1874	0.0179
BAU10_21865	--	capsular biosynthesis protein	2.6390	0.0345
BAU10_21950	--	transcriptional regulator	2.4752	0.0000
BAU10_22015	*kdpD*	DUF48 domain-containing protein	-2.7577	0.0000
BAU10_22060	--	putative secreted calcium-binding protein	2.8672	0.0000
BAU10_22065	*csgD*	helix-turn-helix transcriptional regulator	2.8882	0.0000
BAU10_22085	*yeaC*	MoxR family ATPase	2.0143	0.0000
BAU10_22110	--	hypothetical protein BAU0_220	2.0936	0.0000
BAU10_22220	*yiaW*	DUF3302 domain-containing protein	2.6039	0.0000
BAU10_22345	--	spore coat protein U domain-containing protein	-2.1710	0.0350
BAU10_22360	--	spore coat protein U domain-containing protein	-5.9541	0.0165
BAU10_22405	*phoP*	response regulator transcription factor	-2.0438	0.0133
BAU10_22455	--	membrane protein	-2.5570	0.0000
BAU10_22690	*PA977*	DMT family transporter	-2.5618	0.0000

BAU10_22735	--	RDD family protein	-2.1846	0.0008
BAU10_22765	*nfsB*	oxygen-insensitive NAD(P)H nitroreductase	2.1886	0.0000
BAU10_22775	*cgt*	alpha-amylase	2.1720	0.0000
BAU10_22780	*dacC*	D-alanyl-D-alanine carboxypeptidase	2.2723	0.0017
BAU10_22875	--	hypothetical protein	2.8195	0.0474
BAU10_22895	*yjiG*	membrane protein	-2.0751	0.0000
BAU10_22900	*yjiH*	membrane protein	-2.5781	0.0000
BAU10_23080	--	TRAP transporter small permease	-1036.6667	0.0326
BAU10_23240	*yvcR*	ABC transporter ATP-binding protein	2.2777	0.0001
BAU10_23290	*ahpC*	alkyl hydroperoxide reductase c22 protein	7.0703	0.0000
BAU10_23295	*ahpF*	alkyl hydroperoxide reductase subunit F	7.0011	0.0000
BAU10_23330	--	MFS transporter	-2.0616	0.0000
BAU10_23380	--	hypothetical protein	-2.0597	0.0351
BAU10_23455	--	mechanosensitive ion channel family protein	-2.1578	0.0014
BAU10_23595	--	hypothetical protein	-4.3119	0.0481
BAU10_23660	--	membrane protein	2.0012	0.0000
BAU10_23665	*yhcG*	putative ABC transporter ATP-binding protein	2.5577	0.0000
BAU10_23670	--	putative transcriptional regulator, GntR family protein	2.0357	0.0000
BAU10_23750	--	acyltransferase family protein	-2.7851	0.0000
BAU10_23915	--	lactoylglutathione lyase	2.4745	0.0021
BAU10_24180	*rplJ*	50S ribosomal protein L0	2.1816	0.0000
BAU10_24185	*rplL*	50S ribosomal protein L7/L2	2.1580	0.0000
BAU10_24195	*rpoC*	DNA-directed RNA polymerase beta' subunit	2.0551	0.0000
BAU10_24265	*cadB*	lysine:cadaverine antiporter	5.8960	0.0000
BAU10_24270	*cadA*	lysine decarboxylase LdcC	14.9709	0.0000

Table S4 *Vibrio alginolyticus* proteins are up/down regulated significantly in *rraA* mutant
Orange shade: Differentially expressed prorteins mentioned in the article.

id	Symbol	Description	P-FC	FDR
BAU10_07485	--	tandem-95 repeat protein	-1.7441	0.0000
BAU10_13890	--	ExeM/NucH family extracellular endonuclease	1.7721	0.0000
BAU10_19610	MJECS02	hypothetical protein	3.9522	0.0000
BAU10_19635	Rep	DNA helicase	4.3181	0.0000
BAU10_24250	PurH	bifunctional phosphoribosylaminoimidazolecarboxamide formyltransferase/IMP cyclohydrolase	1.8036	0.0000
BAU10_19625	MJ1519	AAA family ATPase	4.3553	0.0000
BAU10_01460	GrcA	formate acetyl transferase-related protein	2.7816	0.0000
BAU10_19595	--	hypothetical protein	3.7495	0.0000
BAU10_22630	YbiW	formate C-acetyltransferase/glycerol dehydratase family glycyl radical enzyme	1.6690	0.0000
BAU10_19630	--	DEAD/DEAH box helicase	5.8480	0.0000
BAU10_19605	YqhH	DEAD/DEAH box helicase	3.7493	0.0000
BAU10_21165	PgcA	putative phosphomannomutase	1.5919	0.0000
BAU10_23290	AhpC	alkyl hydroperoxide reductase c22 protein	2.3001	0.0000
BAU10_09970	PurF	amidophosphoribosyltransferase	1.5701	0.0000

BAU10_19615	YqhH	DEAD/DEAH box helicase family protein	4.0799	0.0000
BAU10_09875	BdlA	methyl-accepting chemotaxis protein	1.8975	0.0000
BAU10_09250	TsdA	c-type cytochrome	-2.5228	0.0000
BAU10_02355	PurL	phosphoribosylformylglycinamidine synthase	1.7737	0.0000
BAU10_18760	AslA	arylsulfatase	-8.0017	0.0000
BAU10_23405	PutA	bifunctional proline dehydrogenase/L-glutamate gamma-semialdehyde dehydrogenase PutA	-1.9212	0.0000
BAU10_09240	TtrA	tetrathionate reductase subunit TtrA	-1.8996	0.0000
BAU10_19890	YdeP	FdhF/YdeP family oxidoreductase	1.9954	0.0000
BAU10_07495	OprF	putative outer membrane protein	1.6721	0.0000
BAU10_14275	AcsA	acetate--CoA ligase	-3.4526	0.0000
BAU10_17840	NapA	periplasmic nitrate reductase	-2.8279	0.0000
BAU10_18285	PagL	6-phospho-alpha-glucosidase	2.0202	0.0000
BAU10_07105	--	4Fe-4S dicluster domain-containing protein	1.6285	0.0000
BAU10_18570	--	36 domain-containing protein	-3.4938	0.0000
BAU10_04000	ArgT	amino acid ABC transporter, periplasmic amino acid-binding protein	-3.4162	0.0000
BAU10_22340	Cah	carbonic anhydrase	1.9810	0.0000
BAU10_06575	AslA	arylsulfatase A	-9.2755	0.0000
BAU10_07410	AapJ	amino acid ABC transporter, periplasmic amino acid-binding protein	-7.3739	0.0000
BAU10_04925	Ald	alanine dehydrogenase	-3.3230	0.0000
BAU10_07330	Gdh	NAD-glutamate dehydrogenase	-1.6971	0.0000
BAU10_19675	--	ATP-binding protein	4.5993	0.0000
BAU10_03955	YeaG	conserved hypothetical protein	-4.2176	0.0000
BAU10_05315	Hcp	hydroxylamine reductase	4.8154	0.0000
BAU10_05995	VlIY	4-hydroxyphenylpyruvate dioxygenase	-15.3302	0.0000
BAU10_15390	MppA	ABC transporter substrate-binding protein	-2.5219	0.0000
BAU10_13835	--	338 domain-containing protein	-2.9127	0.0000
BAU10_17905	OmpA2	outer membrane protein A	-4.9727	0.0000
BAU10_06570	--	haloacid dehalogenase-like hydrolase	-3.7842	0.0000
BAU10_17045	FusA	elongation factor G	-2.7093	0.0000
BAU10_19380	YeiE	transcriptional regulator, LysR family	2.1174	0.0000
BAU10_21315	--	redoxin family protein	-1.7703	0.0000
BAU10_04735	CitC	citrate carrier	-4.1566	0.0000
BAU10_17235	PurA2	adenylosuccinate synthase	1.5055	0.0000
BAU10_07765	LcrV	VcrV	-1.8111	0.0000
BAU10_05775	PurT	phosphoribosylglycinamide formyltransferase 2	1.9648	0.0000
BAU10_01830	PhoB	DNA-binding response regulator PhoB	-1.5573	0.0000
BAU10_13125	ArgF	Ornithine carbamoyltransferase	-2.8240	0.0000
BAU10_21030	--	tandem-95 repeat protein	-7.5842	0.0000
BAU10_13655	ArgH	argininosuccinate lyase	-1.7790	0.0000
BAU10_20365	YghX	dienelactone hydrolase family protein	-12.0281	0.0000
BAU10_19425	FadL	outer membrane protein transport protein	-1.9397	0.0000
BAU10_13665	ArgB	acetylglutamate kinase	-3.8206	0.0000
BAU10_05930	DapA	dihydrodipicolinate synthase family protein	-15.7119	0.0000
BAU10_20530	ArtI	transporter substrate-binding domain-containing protein	-1.8256	0.0000
BAU10_15555	--	putative hyperosmotically inducible periplasmic protein	-1.7426	0.0000
BAU10_19580	--	hypothetical protein	3.9053	0.0000

BAU10_05605	PurC	phosphoribosylaminoimidazolesuccinocarboxamide synthase	1.7744	0.0000
BAU10_13850	ArgD	bifunctional N-succinyldiaminopimelate-aminotransferase/acetylornithine transaminase protein	-2.3294	0.0000
BAU10_02375	--	succinylglutamate desuccinylase/aspartoacylase family protein	-1.9608	0.0000
BAU10_06495	OusX	L-proline glycine betaine binding ABC transporter protein ProX	-3.8091	0.0000
BAU10_13715	Bfr	bacterioferritin	-2.2358	0.0000
BAU10_20470	PdhA	component subunit alpha	-12.1720	0.0000
BAU10_05985	OppA	peptide ABC transporter substrate-binding protein	-24.4826	0.0000
BAU10_03820	GuaB	inosine monophosphate dehydrogenase-related protein	-2.1065	0.0000
BAU10_22890	--	putative carboxypeptidase G2	-1.6018	0.0000
BAU10_01900	AceA	isocitrate lyase	-4.9185	0.0000
BAU10_04720	OadA1	oxaloacetate decarboxylase alpha subunit	1.6454	0.0000
BAU10_20230	--	protein of avirulence locus	1.7055	0.0000
BAU10_07740	BcsP31	immunogenic protein	-10.2306	0.0000
BAU10_08755	--	imelysin family protein	-4.2013	0.0000
BAU10_11050	--	histidine kinase	-1.8838	0.0000
BAU10_12545	SerA	phosphoglycerate dehydrogenase	1.7301	0.0001
BAU10_20775	YcjY	dienelactone hydrolase	-3.2186	0.0001
BAU10_20625	MmsB	3-hydroxyisobutyrate dehydrogenase	-10.5408	0.0001
BAU10_03950	VP0986	YeaH/YhbH family protein	-5.2404	0.0001
BAU10_20290	PrkC	serine/threonine protein kinase	3.4685	0.0001
BAU10_07125	FdhB1	formate dehydrogenase, iron-sulfur subunit	1.5122	0.0001
BAU10_20285	TagH	type VI secretion system-associated FHA domain protein TagH	3.5839	0.0001
BAU10_18690	HgbA	TonB-dependent siderophore receptor	-32.7301	0.0001
BAU10_19600	--	AAA family ATPase	4.0050	0.0001
BAU10_10505	--	hypothetical protein	-2.0017	0.0001
BAU10_22065	CsgD	helix-turn-helix transcriptional regulator	3.4178	0.0001
BAU10_16450	TnaA	tryptophanase	-2.2862	0.0001
BAU10_13660	ArgG	argininosuccinate synthase	-3.1358	0.0001
BAU10_22015	KdpD	8 domain-containing protein	-20.3388	0.0001
BAU10_14220	AgmR	putative two-component response regulator	-2.1553	0.0002
BAU10_01395	GltB	glutamate synthase large subunit	2.4507	0.0002
BAU10_19585	--	998 domain-containing protein	3.7935	0.0002
BAU10_14480	--	DUF2492 family protein	1.6707	0.0002
BAU10_23570	--	putative amino acid ABC transporter, periplasmic amino acid-binding portion	-3.5284	0.0002
BAU10_22320	HI_1218	putative L-lactate permease	-3.1261	0.0002
BAU10_05555	--	agglutination protein	-2.6131	0.0002
BAU10_06535	EctC	ectoine synthase	-2.6362	0.0003
BAU10_07575	EO	zinc-binding dehydrogenase	-7.9447	0.0003
BAU10_18320	HI_0002	putative long-chain-fatty-acid-CoA ligase	-13.2259	0.0003
BAU10_07695	PrpC	2-methylcitrate synthase	-6.0115	0.0003
BAU10_15270	FadB	fatty acid oxidation complex subunit alpha FadB	-3.1491	0.0003
BAU10_03945	YcgB	SpoVR family protein	-4.8823	0.0003
BAU10_23295	AhpF	alkyl hydroperoxide reductase subunit F	1.8547	0.0004
BAU10_00855	MtlA	PTS mannitol transporter subunit IICBA	-3.0284	0.0004
BAU10_21950	--	transcriptional regulator	1.5788	0.0004
BAU10_10465	PurM	Phosphoribosylformylglycinamidine cyclo-ligase	1.7316	0.0004

BAU10_23920	--	lactoylglutathione lyase	-3.4128	0.0004
BAU10_20955	--	pterin-4-alpha-carbinolamine dehydratase	-2.1494	0.0004
BAU10_03335	FeoB	Fe(2+) transporter permease subunit FeoB	-1.6223	0.0005
BAU10_07750	YopD	type III secretion system translocon subunit VopD	-1.8007	0.0005
BAU10_03770	CysM	cysteine synthase/cystathionine beta-synthase family protein	25.1783	0.0005
BAU10_17830	NapC	periplasmic nitrate reductase, cytochrome c-type protein	-2.8553	0.0006
BAU10_15265	FadA	acetyl-CoA C-acyltransferase FadA	-3.1764	0.0006
BAU10_18435	--	putative acyl-CoA dehydrogenase	-16.1518	0.0006
BAU10_18450	MmsA	methylmalonate-semialdehyde dehydrogenase (acylating)	-18.4429	0.0006
BAU10_00140	--	hypothetical protein	-2.2643	0.0007
BAU10_05455	FadE	acyl-CoA dehydrogenase	-2.7104	0.0007
BAU10_14885	PurK	5-(carboxyamino)imidazole ribonucleotide synthase	1.8527	0.0007
BAU10_19765	--	late competence development ComFB family protein	-2.7574	0.0007
BAU10_05905	AldH	aldehyde dehydrogenase (NADP(+))	-21.9253	0.0007
BAU10_22900	YjiH	membrane protein	-1.9265	0.0008
BAU10_08570	VP1870	TIGR01620 family protein	-2.1882	0.0008
BAU10_03995	AotP	amino acid ABC transporter, ATP-binding protein	-2.2322	0.0008
BAU10_17355	--	aminotransferase, classes I and II superfamily	-2.0769	0.0008
BAU10_05920	--	spermidine/putrescine ABC transporter substrate-binding protein	-32.8377	0.0008
BAU10_07475	--	agglutination protein	-3.5161	0.0008
BAU10_19010	NemA	alkene reductase	-3.6377	0.0009
BAU10_06930	LuxO	sigma-54-dependent Fis family transcriptional regulator	-4.9724	0.0009
BAU10_04045	--	porin, putative	-1.9441	0.0009
BAU10_14250	FumC2	aspartate ammonia-lyase	-2.8060	0.0009
BAU10_15385	GsiA	peptide ABC transporter, ATP-binding protein	-2.2631	0.0010
BAU10_06615	FlcR	DeoR/GlpR transcriptional regulator	-2.3687	0.0010
BAU10_09230	TtrB	4Fe-4S dicluster domain-containing protein	-2.0327	0.0012
BAU10_06505	OusV	ABC superfamily (glycine/betaine/proline transport protein)	-3.2898	0.0012
BAU10_20535	ArtP	arginine ABC transporter ATP-binding protein ArtP	-1.7773	0.0012
BAU10_21380	FdhA	formate dehydrogenase subunit alpha	1.6571	0.0012
BAU10_23225	--	DUF302 domain-containing protein, partial	-7.0318	0.0012
BAU10_22010	KdpE	DNA-binding response regulator	-12.1330	0.0013
BAU10_17495	YjjU	patatin family protein	-1.7107	0.0013
BAU10_05960	APP2	aminopeptidase P family protein	-5.7270	0.0013
BAU10_20475	PdhB	component, beta subunit	-22.7159	0.0014
BAU10_20770	YcjY	putative dienelactone hydrolase-related enzyme	-2.0327	0.0016
BAU10_16345	RimK	ribosomal protein S6 modification protein	-3.6013	0.0016
BAU10_15965	BtuB	TonB-dependent receptor	-3.5821	0.0016
BAU10_19590	--	DUF2075 domain-containing protein	-0.2509	0.0016
BAU10_06190	DhkJ	hybrid sensor histidine kinase/response regulator	-18.2082	0.0016
BAU10_00635	Mdh	malate dehydrogenase	-1.5483	0.0016
BAU10_20640	Acad8	acyl-CoA dehydrogenase	-14.6219	0.0017
BAU10_20645	MmsA	CoA-acylating methylmalonate-semialdehyde dehydrogenase	-13.3064	0.0018
BAU10_19620	--	hypothetical protein	4.3290	0.0023
BAU10_17115	--	hypothetical protein	-1.7344	0.0027
BAU10_20500	HSP31	glutamine amidotransferase domain-containing protein	-1.8183	0.0028
BAU10_16405	--	TAXI family TRAP transporter solute-binding subunit	-2.9721	0.0031

BAU10_18445	ThlA	thiolase family protein	-7.4099	0.0032
BAU10_08860	--	glycine cleavage system regulatory protein	-2.3548	0.0033
BAU10_24245	PurD	phosphoribosylamine--glycine ligase	1.6200	0.0033
BAU10_18335	--	ABC transporter substrate-binding protein	-17.7668	0.0034
BAU10_23400	--	1-pyrroline-5-carboxylate dehydrogenase	-2.1500	0.0034
BAU10_22775	Cgt	alpha-amylase	-0.3300	0.0035
BAU10_07935	--	putative type III export apparatus protein NosA	-2.2118	0.0038
BAU10_23740	--	putative inner membrane protein	-2.0627	0.0039
BAU10_02075	YrbD	sodium/alanine symporter	-4.3789	0.0040
BAU10_05260	MdtA	putative periplasmic linker protein	-2.1790	0.0044
BAU10_06265	Sll1178	hypothetical protein	-1.6318	0.0045
BAU10_13930	MotX	sodium-type polar flagellar protein MotX	-1.8104	0.0045
BAU10_06935	--	Hpt domain-containing protein	-3.3227	0.0047
BAU10_16310	--	putative outer membrane protein	-3.7629	0.0050
BAU10_07480	Pal	putative outer membrane protein	-6.6243	0.0051
BAU10_20095	CtpH	methyl-accepting chemotaxis protein	-3.5672	0.0052
BAU10_20810	SAR0107	putative AraC-type regulatory protein	1.6362	0.0056
BAU10_15210	IbpA	16 kDa heat shock protein A	-2.1338	0.0056
BAU10_16250	--	energy transducer TonB	-1.6298	0.0057
BAU10_01540	ZraR	sigma-54 dependent transcriptional regulator	1.9756	0.0058
BAU10_18525	AF_1420	putative stomatin-like protein	-3.1418	0.0065
BAU10_07690	AcnD	Fe/S-dependent 2-methylisocitrate dehydratase AcnD	-2.5255	0.0065
BAU10_20485	EtfA	electron transfer flavoprotein subunit alpha	-46.3587	0.0071
BAU10_05910	VP1330	4-hydroxyproline epimerase	-23.1912	0.0073
BAU10_20820	PstS1	ABC transporter, periplasmic substrate-binding protein	-5.7650	0.0074
BAU10_23895	EmrK	HlyD family secretion protein	2.3888	0.0078
BAU10_15975	YejE	putative peptide ABC transporter, permease protein	26.4426	0.0083
BAU10_22395	SodC	superoxide dismutase	-4.5901	0.0089
BAU10_19370	VPA0850	YceI family protein	-1.6565	0.0089
BAU10_18475	CLKR27	3-oxoacyl-ACP reductase	-41.3703	0.0090
BAU10_07680	PA3568	propionyl-CoA synthetase	-5.3911	0.0092
BAU10_18455	ACAD8	acyl-CoA dehydrogenase	-7.5307	0.0093
BAU10_07805	--	type III secretion system gatekeeper subunit SctW	-8.0832	0.0093
BAU10_19415	--	YgdI/YgdR family lipoprotein	-8.8876	0.0093
BAU10_11100	FadD15	AMP-binding protein	-5.3840	0.0102
BAU10_22325	lldD	L-lactate dehydrogenase (cytochrome)	-2.1363	0.0104
BAU10_03780	--	D-Tyr-tRNAtyr deacylase	-1.5514	0.0104
BAU10_05255	--	efflux RND transporter periplasmic adaptor subunit	-1.6779	0.0108
BAU10_06865	--	porin family protein	-3.1478	0.0108
BAU10_03130	--	putative nitrogen regulatory protein P-II family protein	-2.1618	0.0109
BAU10_23690	--	family protein	-1.7231	0.0111
BAU10_08765	--	peptidase	-1.5271	0.0111
BAU10_02280	RecN	DNA repair protein RecN	1.8971	0.0117
BAU10_19420	VolA	lipase	-1.6132	0.0119
BAU10_05925	LhpI	ornithine cyclodeaminase family protein	-27.2307	0.0122
BAU10_09545	--	ABC transporter substrate-binding protein	-3.6896	0.0123
BAU10_16330	SpeA	arginine decarboxylase	-2.6041	0.0123

BAU10_03595	DctP	TRAP-type C4-dicarboxylate transport system component	-2.5898	0.0123
BAU10_15060	PheC	transporter substrate-binding domain-containing protein	-2.7494	0.0144
BAU10_22005	KtrA	potassium transporter TrkA	-69.5120	0.0145
BAU10_15710	PA1727	EAL domain-containing protein	-1.5150	0.0146
BAU10_02980	FlgB	flagellar basal-body rod protein B	-2.2633	0.0157
BAU10_15470	YgbN	low-affinity gluconate/H+ symporter GntU	-1.6767	0.0159
BAU10_19055	--	repeat family protein	-1.5385	0.0167
BAU10_01875	PA2428	polyphosphate kinase 2	-2.0195	0.0168
BAU10_20390	SdaC	HAAAP family serine/threonine permease	-4.5495	0.0168
BAU10_10345	FlaB	flagellin	-1.5178	0.0168
BAU10_01660	HI_1126.1	putative carbon starvation protein A	-1.6803	0.0172
BAU10_07700	PrpB	carboxyphosphonoenolpyruvate phosphonomutase	-2.9005	0.0172
BAU10_07585	GlnQ	amino acid ABC transporter ATP-binding protein	-1.5427	0.0172
BAU10_09280	OppA	ABC transporter substrate-binding protein	-1.8582	0.0172
BAU10_09125	Isoc1	isochorismatase family protein	-4.5149	0.0175
BAU10_21660	HutZ	heme utilization protein HutZ	-1.8607	0.0185
BAU10_20320	--	putative glutathione S-transferase	-1.7590	0.0186
BAU10_07880	--	type III chaperone	-2.3297	0.0192
BAU10_19695	--	hypothetical protein	-0.2590	0.0193
BAU10_23915	--	lactoylglutathione lyase	-1.6184	0.0193
BAU10_05560	Csd	cysteine desulfurase-like protein	-1.9288	0.0196
BAU10_14190	SodA	superoxide dismutase, Mn	-3.0809	0.0201
BAU10_05665	HutG	formimidoylglutamase	-2.8108	0.0203
BAU10_16245	ExbD2	TonB system transport protein ExbD2	-1.9459	0.0203
BAU10_14730	Ggt	gamma-glutamyltransferase	-1.5847	0.0206
BAU10_23745	NhaD	sodium:proton antiporter NhaD	-4.4925	0.0213
BAU10_07895	YscJ	putative type III secretion lipoprotein	-3.0359	0.0221
BAU10_21785	FatB	siderophore ABC transporter substrate-binding protein	-15.8201	0.0227
BAU10_08760	--	c-type cytochrome	-1.5903	0.0229
BAU10_05660	HutU	urocanate hydratase	-1.5046	0.0238
BAU10_08470	KilA	DNA-binding protein	1.8854	0.0239
BAU10_07565	ArtI	nickel transporter	-4.6547	0.0239
BAU10_19080	PldA	putative outer membrane phospholipase A precursor	-1.6917	0.0240
BAU10_00850	MtlD	mannitol-1-phosphate 5-dehydrogenase	-2.5264	0.0243
BAU10_11095	--	Outer membrane receptor for Fe^{3+}-dicitrate	-8.4099	0.0245
BAU10_07875	VopS	Va1686	-2.5539	0.0255
BAU10_10085	FadI	acetyl-CoA C-acyltransferase FadI	-1.6720	0.0257
BAU10_00485	CysG	uroporphyrinogen-III C-methyltransferase	-3.0070	0.0260
BAU10_16465	HI_0519	nucleoside permease NupC	-4.2488	0.0262
BAU10_20490	PA2953	electron transfer flavoprotein-ubiquinone oxidoreductase	-3.3567	0.0274
BAU10_13840	AstD	succinylglutamate 5-semialdehyde dehydrogenase	-2.1715	0.0274
BAU10_09745	--	SDR family oxidoreductase	-4.7444	0.0274
BAU10_02405	Crl	transcriptional regulator Crl	-2.1408	0.0276
BAU10_12030	VP2494	YacL family protein	-2.3505	0.0277
BAU10_23890	OmpW	outer membrane protein OmpW	-1.6112	0.0282
BAU10_03000	FlgF	polar flagellar FlgF	-2.4354	0.0292
BAU10_14970	YihG	acyltransferase	-1.5504	0.0298

BAU10_23575	DhkK	hybrid sensor histidine kinase/response regulator	-4.5388	0.0321
BAU10_07110	TorD	putative formate dehydrogenase-specific chaperone	1.5704	0.0326
BAU10_13200	--	LPS export ABC transporter periplasmic protein LptC	-1.5219	0.0326
BAU10_20635	YngF	enoyl-CoA hydratase	-22.1291	0.0336
BAU10_13275	YlaK	PhoH family protein	-3.2950	0.0336
BAU10_17260	DoeD	aspartate aminotransferase family protein	-3.4412	0.0344
BAU10_18460	PaaF	enoyl-CoA hydratase	-9.2620	0.0344
BAU10_12815	DavD	NAD-dependent succinate-semialdehyde dehydrogenase	1.6731	0.0357
BAU10_19330	Gph	HAD-IA family hydrolase	-1.5901	0.0376
BAU10_14610	HemD	uroporphyrinogen-III synthase	-1.5015	0.0387
BAU10_09065	--	EAL domain-containing protein	-4.0931	0.0391
BAU10_14265	--	cyclic nucleotide-binding protein	-3.8032	0.0438
BAU10_18305	EtfB	electron transfer flavoprotein, beta-subunit	-21.9486	0.0473
BAU10_11780	--	hypothetical protein	-1.8604	0.0477
BAU10_10875	RpoE	putative RNA polymerase ECF-type sigma factor	-2.9894	0.0477
BAU10_18970	CalB	coniferyl aldehyde dehydrogenase	-1.7347	0.0478
BAU10_05965	OppF	ATP-binding cassette domain-containing protein	-12.5670	0.0488
BAU10_23260	--	GNAT family N-acetyltransferase	-1.9485	0.0490
BAU10_06000	VC_1345	1,2-dioxygenase	-11.0675	0.0491
BAU10_23065	RhmA	aldolase	-37636.7733	0.0493
